# Functional Variants in *NFKBIE* and *RTKN2* Involved in Activation of the NF-κB Pathway Are Associated with Rheumatoid Arthritis in Japanese

**DOI:** 10.1371/journal.pgen.1002949

**Published:** 2012-09-13

**Authors:** Keiko Myouzen, Yuta Kochi, Yukinori Okada, Chikashi Terao, Akari Suzuki, Katsunori Ikari, Tatsuhiko Tsunoda, Atsushi Takahashi, Michiaki Kubo, Atsuo Taniguchi, Fumihiko Matsuda, Koichiro Ohmura, Shigeki Momohara, Tsuneyo Mimori, Hisashi Yamanaka, Naoyuki Kamatani, Ryo Yamada, Yusuke Nakamura, Kazuhiko Yamamoto

**Affiliations:** 1 Laboratory for Autoimmune Diseases, Center for Genomic Medicine (CGM), RIKEN, Yokohama, Japan; 2 Department of Allergy and Rheumatology, Graduate School of Medicine, the University of Tokyo, Tokyo, Japan; 3 Laboratory for Statistical Analysis, CGM, RIKEN, Yokohama, Japan; 4 Center for Genomic Medicine, Kyoto University Graduate School of Medicine, Kyoto, Japan; 5 Department of Rheumatology and Clinical Immunology, Graduate School of Medicine, Kyoto University, Kyoto, Japan; 6 Institute of Rheumatology, Tokyo Women's Medical University, Tokyo, Japan; 7 Laboratory for Medical Informatics, CGM, RIKEN, Yokohama, Japan; 8 Laboratory for Genotyping Development, CGM, RIKEN, Yokohama, Japan; 9 CREST Program, Japan Science and Technology Agency, Kawaguchi, Saitama, Japan; 10 Institut National de la Sant? et de la Recherche M?dicale (INSERM), Unit? U852, Kyoto University Graduate School of Medicine, Kyoto, Japan; 11 Laboratory for International Alliance, CGM, RIKEN, Yokohama, Japan; 12 Unit of Statistical Genetics, Center for Genomic Medicine, Graduate School of Medicine, Kyoto University, Kyoto, Japan; 13 Laboratory of Molecular Medicine, Human Genome Center, Institute of Medical Science, University of Tokyo, Tokyo, Japan; The Wellcome Trust Sanger Institute, United Kingdom

## Abstract

Rheumatoid arthritis is an autoimmune disease with a complex etiology, leading to inflammation of synovial tissue and joint destruction. Through a genome-wide association study (GWAS) and two replication studies in the Japanese population (7,907 cases and 35,362 controls), we identified two gene loci associated with rheumatoid arthritis susceptibility (*NFKBIE* at 6p21.1, rs2233434, odds ratio (OR) = 1.20, *P* = 1.3×10^−15^; *RTKN2* at 10q21.2, rs3125734, OR = 1.20, *P* = 4.6×10^−9^). In addition to two functional non-synonymous SNPs in *NFKBIE*, we identified candidate causal SNPs with regulatory potential in *NFKBIE* and *RTKN2* gene regions by integrating *in silico* analysis using public genome databases and subsequent *in vitro* analysis. Both of these genes are known to regulate the NF-κB pathway, and the risk alleles of the genes were implicated in the enhancement of NF-κB activity in our analyses. These results suggest that the NF-κB pathway plays a role in pathogenesis and would be a rational target for treatment of rheumatoid arthritis.

## Author Summary

Rheumatoid arthritis (RA) is a chronic autoimmune disease affecting approximately 1% of the general adult population. More than 30 susceptibility loci for RA have been identified through genome-wide association studies (GWAS), but the disease-causal variants at most loci remain unknown. Here, we performed replication studies of the candidate loci of our previous GWAS using Japanese cohorts and identified variants in *NFKBIE* and *RTKN2* gene loci that were associated with RA. To search for causal variants in both gene regions, we first examined non-synonymous (ns)SNPs that alter amino-acid sequences. As *NFKBIE* and *RTKN2* are known to be involved in the NF-κB pathway, we evaluated the effects of nsSNPs on NF-κB activity. Next, we screened *in silico* variants that may regulate gene transcription using publicly available epigenetic databases and subsequently evaluated their regulatory potential using *in vitro* assays. As a result, we identified multiple candidate causal variants in *NFKBIE* (2 nsSNPs and 1 regulatory SNP) and *RTKN2* (2 regulatory SNPs), indicating that our integrated *in silico* and *in vitro* approach is useful for the identification of causal variants in the post–GWAS era.

## Introduction

Rheumatoid arthritis (RA [MIM 180300]) is an autoimmune disease [1] with a complex etiology involving several genetic factors as well as environmental factors. Previous genome-wide association studies (GWAS) for RA have discovered many genetic loci [2]–[6], although the causal mechanisms linking the variants in these loci and disease etiology are largely unknown, except for in a few cases [6]–[8]. In contrast to mutations in Mendelian, monogenic diseases, most disease-associated variants in complex diseases, including autoimmune diseases, have moderate effects on disease susceptibility. This is because the disease causal variants in complex diseases are thought to have moderate effects on gene function, while amino acid changes introduced by the mutations of monogenic diseases have critical impacts on protein function [9]. Moreover, it has been demonstrated that the majority of autoimmune disease loci are expression quantitative trait loci (eQTLs) [10], [11], indicating that accumulation of quantitative, but not qualitative, changes in gene function likely predisposes individuals to the disease. This renders it difficult to pinpoint the causal variants in the GWAS loci, especially in eQTLs, because all the variations in strong linkage disequilibrium (LD) with the marker SNP in a GWAS, the majority of which are not covered by the SNP array, are possible candidates for causal variants.

In recent years, with the emergence of next-generation sequencing technologies, the way we approach disease-causing variants has dramatically changed. First, a comprehensive map of human genetic variations is now available owing to the 1000 Genome Project [12], which allows us to grasp most of the potential common variants. This also enables us to perform genotype imputation of SNPs that are not directly genotyped in the GWAS, and consequently, to test them for association. Second, genomic studies using new technologies, such as chromatin immunoprecipitation-sequencing (ChIP-seq) and DNase I hypersensitive sites sequencing (DNase-seq), have advanced our understanding of how each genomic cluster regulates gene transcription. If disease-associated variants are present in a critical site for gene regulation suggested by the ChIP-seq and DNase-seq studies, the disease-associated variants might possibly influence gene transcription levels such as through altered transcription factor-DNA binding avidity.

In the present study, we first performed replication studies of candidate loci in our previous GWAS and identified two association signals with genome-wide significance (*P*<5×10^−8^) in nuclear factor of kappa light polypeptide gene enhancer in B-cells inhibitor, epsilon (*NFKBIE* [MIM 604548]) and rhotekin 2 (*RTKN2*) loci. By utilizing publicly available datasets yielded by the above-mentioned genomic studies, we then performed integrated *in silico* and *in vitro* analysis to identify plausible causal variants in *NFKBIE* and *RTKN2* loci.

## Results

### Identification of rheumatoid arthritis susceptibility genes

**Figure 1:**
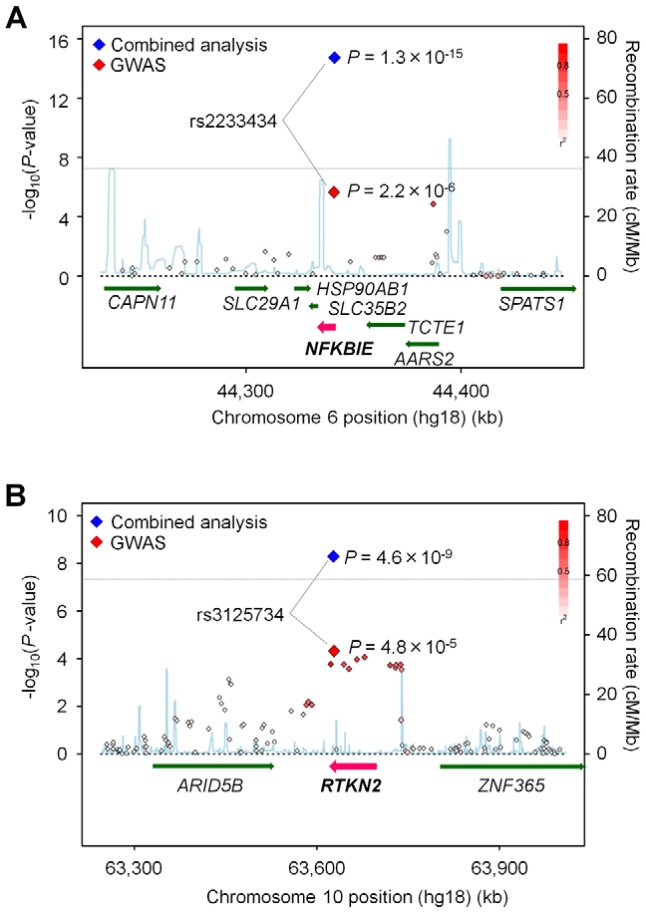
Association plots of *NFKBIE* and *RTKN2* regions. The diamonds represent the -log_10_ of the Cochran-Armitage trend *P*-values. Large diamonds show landmark SNPs in *NFKBIE* (rs2233434: A) and *RTKN2* (rs3125734: B). Red: GWAS, Blue: combined analysis. Red colors of each SNP indicate its *r^2^* with landmark SNP. Gray lines indicate the genome-wide significance threshold (*P*<5×10^−8^). For each plot, the -log_10_ *P*-values (y-axis) of the SNPs are presented according to their chromosomal positions (x-axis). Physical positions are based on NCBI build 36.3 of the human genome. Genetic recombination rates, estimated using the 1000 Genome Project (JPT and CHB), are represented by the blue line.

We previously performed a GWAS of RA using a Japanese case-control cohort (2,303 cases and 3,380 controls) and identified significant associations in major histocompatibility complex, class II, DR beta 1 (*HLA-DRB1* [MIM 142857]), and chemokine (C-C motif) receptor 6 (*CCR6* [MIM 601835]) loci (*P*_GWAS_<5×10^−8^) [6]. To reveal additional risk loci from those showing moderate associations in the GWAS (31 loci, 5×10^−8^<*P*_GWAS_ *<*5×10^−5^), we selected a landmark SNP from each locus and genotyped it for an additional cohort (replication-1: 2,187 cases and 28,219 controls) (Table S1, S2). Among the 31 SNPs genotyped, seven SNPs were nominally associated with RA (*P*<0.05), which included SNPs in the tumor necrosis factor, alpha-induced protein 3 (*TNFAIP3* [MIM 191163]), and signal transducer and the activator of transcription 4 (*STAT4* [MIM 600558]) gene loci that were previously reported to be associated with RA [13], [14] (Table S2). In a combined analysis of the GWAS and the 1st replication study, we identified two associations with genome-wide significance (*P*<5×10^−8^) in *NFKBIE* (6p21.1, rs2233434, *P* = 4.1×10^−11^, odds ratio (OR) = 1.21, 95% confidence interval (CI) = 1.14–1.28) and in *RTKN2* (10q21.2, rs3125734, *P* = 3.7×10^−8^, OR = 1.23, 95% CI = 1.14–1.32) (Table 1 and Figure 1). *NFKBIE* was previously reported as a novel RA susceptibility gene locus in a meta-analysis of three GWASs for RA in the Japanese population, which included the GWAS set that the present study used [15]. *RTKN2* is located in the same region (10q21) as *ARID5B*, in which a significant association signal was also reported in the meta-analysis [15]. In our GWAS set, however, two significant signals were observed at rs3125734 (*RTKN2*: *P* = 4.8×10^−5^) and rs10821944 (*ARID5B*: *P* = 7.4×10^−4^), the former of which was tested as a landmark in the replication study. These two SNPs were in weak LD (*r^2^* = 0.11) and the independent effect of each SNP was observed after conditioning on each SNP (*RTKN2*: *P* = 1.5×10^−3^, *ARID5B*: *P* = 0.024, respectively). This indicated that two independent associations existed in this region, and the association of *RTKN2* is novel. We also confirmed the association in the *STAT4* locus [14] with genome-wide significance (2q32.2, rs10168266, *P* = 3.2×10^−8^, OR = 1.16, 95% CI = 1.10–1.22) (Table S2). The associations in *NFKBIE* and *RTKN2* were further replicated in the 2nd replication cohort (3,417 cases and 3,763 controls; rs2233434, *P* = 1.1×10^−5^, OR = 1.19, 95% CI = 1.10–1.30 and rs3125734, *P* = 0.016, OR = 1.14, 95% CI = 1.02–1.26, respectively), confirming the associations in these loci (a combined analysis of three sets; rs2233434, *P* = 1.3×10^−15^, OR = 1.20, 95% CI = 1.15–1.26 and rs3125734, *P* = 4.6×10^−9^, OR = 1.20, 95% CI = 1.13–1.27, respectively) (Table 1 and Figure 1). We also genotyped these SNPs for individuals with systemic lupus erythematosus (SLE [MIM 152700]) (*n* = 657) and Graves' disease (GD [MIM 275000]) (*n* = 1,783). We identified a significant association of *RTKN2* (rs3125734) with GD (*P* = 3.4×10^−5^, OR = 1.24, 95% CI = 1.12–1.37), whereas no significant associations were detected in *NFKBIE* (rs2233434) with either disease or in *RTKN2* (rs3125734) with SLE (Table S3).

**Table 1:** Association analysis of *NFKBIE* and *RTKN2* with rheumatoid arthritis.

		Allele		Number of subjects		Frequency of allele 1			
Gene	dbSNP ID	(1/2)	Study set	Case	Control	Case	Control	Odds ratio (95% CI)	*P*-value^a^
*NFKBIE*	rs2233434	G/A	GWAS	2,303	3,380	0.254	0.216	1.24 (1.13–1.35)	2.2×10^−6^
			Replication study-1	2,186	28,204	0.245	0.215	1.19 (1.10–1.27)	4.2×10^−6^
			Replication study-2	3,396	3,756	0.239	0.209	1.19 (1.10–1.30)	1.1×10^−5^
			Combined analysis	7,885	35,340	0.245	0.215	1.20 (1.15–1.26)	1.3×10^−15^
*RTKN2*	rs3125734	T/C	GWAS	2,303	3,380	0.125	0.101	1.27 (1.13–1.43)	4.8×10^−5^
			Replication study-1	2,185	28,218	0.129	0.110	1.20 (1.09–1.31)	1.4×10^−4^
			Replication study-2	3,402	3,751	0.115	0.103	1.14 (1.02–1.26)	0.016
			Combined analysis	7,890	35,349	0.122	0.108	1.20 (1.13–1.27)	4.6×10^−9^

a : Cochran-Armitage trend test was used for the GWAS and replication studies. Mantel-Haenszel method was used for the combined analysis.

### Functional analysis of non-synonymous SNPs

**Figure 2:**
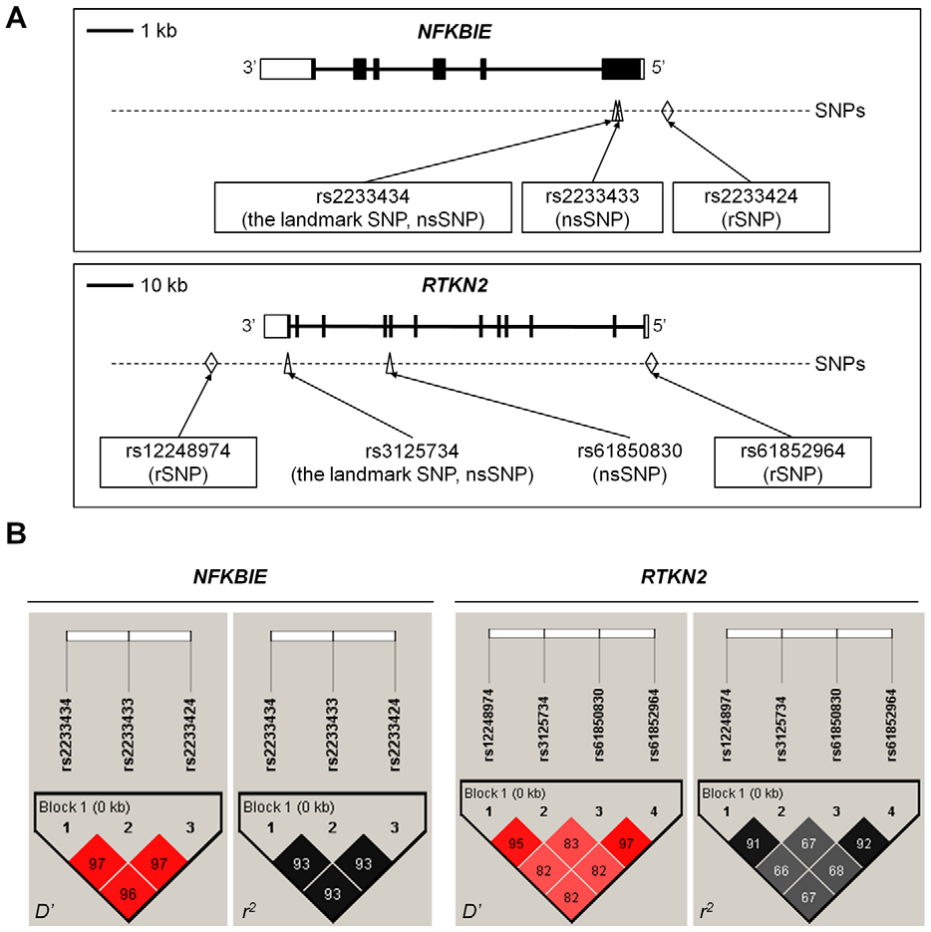
Genomic position and LD blocks. (A) Genomic position of non-synonymous (ns)SNPs and regulatory (r)SNPs in *NFKBIE* and *RTKN2*. *NFKBIE* (top) and *RTKN2* (bottom) correspond to transcripts NM_004556.2 and NM_145307.2, respectively. Exons are shown as boxes, where black boxes represent coding regions and open boxes represent untranslated regions. Intron sequences are drawn as lines. Open triangles represents nsSNPs and open diamond shapes indicate candidate rSNPs. dbSNP IDs of candidate causal variants were boxed in a solid line. (B) LD patterns for nsSNPs and candidate rSNPs in *NFKBIE* (left) and *RTKN2* (right) gene regions. LD blocks were constructed from genotype data of 3,290 control individuals of the GWAS. The diagrams show pairwise LD values as quantified using the *D′* and *r^2^* values.

*NFKBIE* and *RTKN2* genes are both involved in the NF-κB pathway: *NFKBIE* encodes IκB epsilon (IκBε), a member of the IκB family [16], and its binding to NF-κB inhibits the nuclear translocation of NF-κB [17]; *RTKN2* encodes a member of Rho-GTPase effector proteins highly expressed in CD4^+^ T cells [18] and is implicated in the activation of the NF-κB pathway [19]. Considering that the NF-κB pathway is critical for the pathogenesis of RA [20], these two genes could be strong candidates in these regions. To identify disease-causing variants, we first sequenced the coding regions of the genes using DNA from patients (*n* = 48) to find variants that alter amino acid sequences. We identified four non-synonymous (ns)SNPs, which were all registered in the dbSNP database: two nsSNPs in *NFKBIE* (rs2233434 (Val194Ala) and rs2233433 (Pro175Leu)) and two in *RTKN2* (rs3125734 (Arg462His) and rs61850830 (Ala288Thr)), where rs2233434 and rs3125734 were the same as the landmark SNPs in the GWAS (Figure 1 and Figure 2A). The two nsSNPs of each locus were in strong LD (Figure 2B) and were both associated with disease (Table S4). In the haplotype analysis, a single common risk haplotype with a frequency >0.05 was observed in each locus, and significant associations with disease risk were detected (*NFKBIE*, *P* = 5.3×10^−8^, Table S5; *RTKN2*, *P* = 5.7×10^−5^, Table S6).

**Figure 3:**
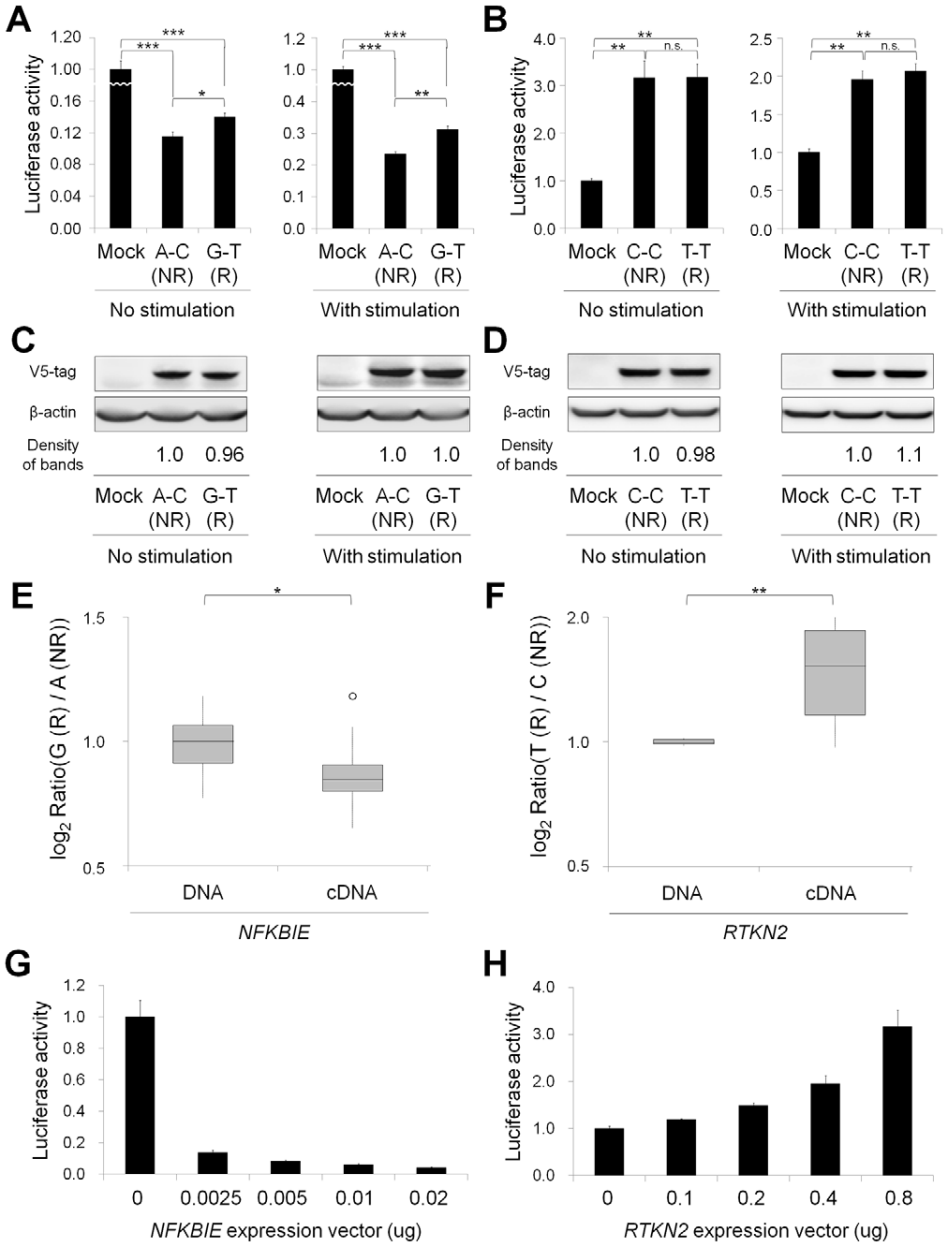
Functional evaluation of nsSNPs and allelic imbalance of expression in *NFKBIE* and *RTKN2*. (A, B) Effects of nsSNPs in *NFKBIE* (A) and *RTKN2* (B) on NF-κB activity by luciferase assays. Two haplotype constructs (A-C (rs2233434-rs2233433; non-risk (NR)) and G-T (risk (R)) for *NFKBIE* and C-C (rs3125734-rs61850830; NR) and T-T (R) for *RTKN2*) were used. The expression vector of each construct, pGL4.32[*luc2P*/NF-κB-RE] vector and pRL-TK vector were transfected into HEK293A cells. Data represent the mean ± s.d. Each experiment was performed in sextuplicate, and experiments were independently repeated three times. *P<0.05, **P<1.0×10^−5^, and ***P<1.0×10^−10^ by Student's *t*-test. n.s.: not significant. (C, D) Protein expression levels of each haplotype construct. Anti-V5 tag antibody was used in the Western blotting analysis to detect the expression of exogenous IκBε (C) and RTKN2 (D). Beta-actin expression was used as an internal control. The densities of the bands were quantified and normalized to that of the risk allele. (E, F) Allelic imbalance of expression in *NFKBIE* (E) and *RTKN2* (F). ASTQ was performed using samples from individuals heterozygous for rs2233434 (G/A) in *NFKBIE* and rs3125734 (T/C) in *RTKN2*. Genomic DNAs and cDNAs were extracted from PBMCs (*n* = 14 for *NFKBIE* and *n* = 6 for *RTKN2*). The y-axis shows the log_2_ ratio of the transcript amounts in target SNPs (risk allele/non-risk allele). The top bar of the box-plot represents the maximum value and the lower bar represents the minimum value. The top of box is the third quartile, the bottom of box is the first quartile, and the middle bar is the median value. The circle is an outlier. *P = 0.012, **P = 0.016, by Student's *t*-test. (G, H) Dose-dependent inhibition of *NFKBIE* (G) and activation of *RTKN2* (H) on NF-κB activity. Various doses of expression vectors carrying the non-risk allele of each gene were transfected into HEK293A cells with pGL4.32 and pRL-TK vectors.

To investigate the effect of these nsSNPs on protein function, we evaluated them by *in silico* analysis using PolyPhen and SIFT software, which predicts possible impacts of amino acid substitutions on the structure and function of proteins, but all four nsSNPs were predicted to have little effect (Table S7), contrasting with the effect of Mendelian disease mutations [9]. We next examined their influence on the NF-κB activity in cells by performing NF-κB reporter assays with haplotype-specific expression vectors. In *NFKBIE*, the non-risk haplotype (A-C: rs2233434 (non-risk allele (NR))-rs2233433 (NR)) displayed an inhibitory effect on NF-κB activity compared with the mock construct, which reflected compulsorily binding of exogenous IκBε to the endogenous NF-κB, as shown in a previous study [16]. Of note, the risk haplotype (G-T: risk allele (R)-R) showed higher NF-κB activity than A-C (NR-NR) (Figure 3A), suggesting impaired inhibitory potential of G-T (R-R) products. No haplotypic difference was detected in the protein expression levels of these constructs (Figure 3C). We also examined two additional constructs of G-C (R-NR) and A-T (NR-R) haplotypes to evaluate the effect of each nsSNP (Figure S1A, S1B). Because NF-κB activity increased in the order of A-C<G-C<A-T<G-T (rs2233434-rs2233433: NR-NR<R-NR<NR-R<R-R) when cells were stimulated with TNF-α, the C>T substitution (Pro175Leu) in rs2233433 may have more impact on the protein function of IκBε compared with the A>G substitution (Val194Ala) in rs2233434. In contrast to the observations in *NFKBIE*, no clear difference was detected between the two common haplotype products of *RTKN2* in either their effect on NF-κB activity or protein expression levels, although both products enhanced NF-κB activity as reported previously (Figure 3B, 3D) [19]. These functional analyses of nsSNPs suggest that two nsSNPs (rs2233434 and rs2233433) in the *NFKBIE* region are candidates for causal SNPs.

### ASTQ analysis suggested the existence of regulatory variants

As the majority of autoimmune disease loci have been implicated as eQTL [11], we speculated that variants in the *NFKBIE* and *RTKN2* loci would influence gene function by regulating gene expression, in addition to changing the amino acid sequences. To address this possibility, we performed allele**-**specific transcript quantification (ASTQ) analysis by using allele-specific probes targeting the nsSNPs in exons (rs2233434 for *NFKBIE* and rs3125734 for *RTKN2*, both of which were the GWAS landmarks). The genomic DNAs and cDNAs were extracted from peripheral blood mononuclear cells (PBMCs) in individuals with heterozygous genotype (*n* = 14 for *NFKBIE* and *n* = 6 for *RTKN2*) and from lymphoblastoid B-cell lines (*n* = 9) for *NFKBIE*. As the expression levels of *RTKN2* were low in lymphoblastoid B cells, only PBMCs were used. When quantified by allele-specific probes, transcripts from the risk allele of *NFKBIE* showed 1.1-fold and 1.2-fold lower amounts (in PBMCs and lymphoblastoid B cells, respectively) than those from non-risk alleles (*P* = 0.012 and 5.3×10^−4^, respectively; Figure 3E and Figure S2). In contrast, 1.5-fold higher amounts of transcripts were observed in the risk allele of *RTKN2* (*P* = 0.016; Figure 3F). These allelic imbalances suggested that both gene loci were eQTL and that there existed variants with *cis*-regulatory effects. Moreover, considering the inhibitory effects of *NFKBIE* and the activating potential of *RTKN2* on NF-κB activity, which might both be dose dependent (Figure 3G, 3H), these regulatory variants in the risk alleles should enhance NF-κB activity *in vivo*.

### Integrated *in silico* and *in vitro* analysis to search for regulatory variants

**Figure 4:**
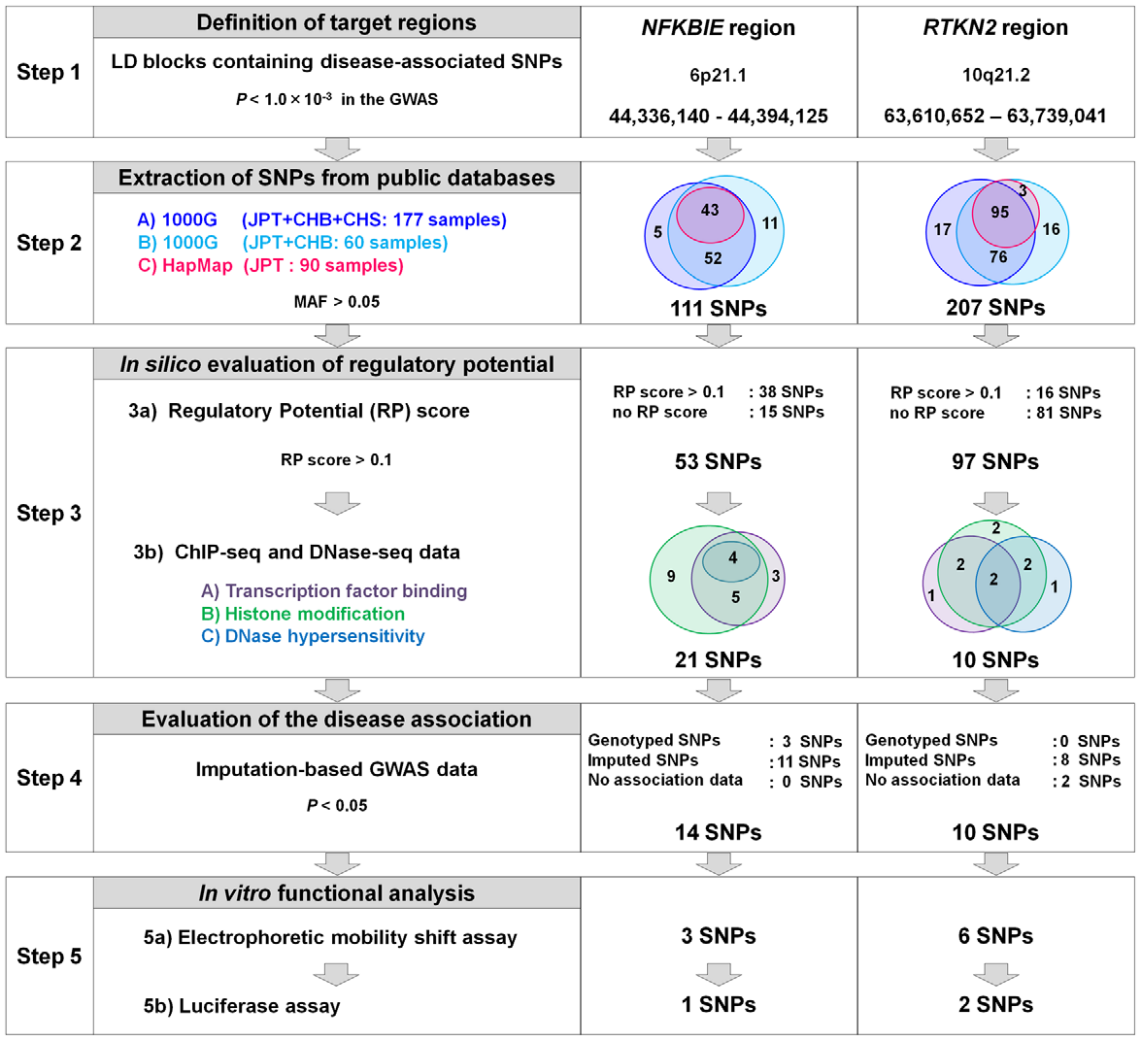
Overview of SNP selection using integrated *in silico* and *in vitro* approaches. The figure shows the SNP selection process (left) and the results of *NFKBIE* (middle) and *RTKN2* (right). (Step 1) LD blocks that contain disease-associated SNPs (*P*_GWAS_<1.0×10^−3^) were selected. (Step 2) SNPs were extracted from three databases (A–C). 1000G, 1000 Genome Project; HapMap, International HapMap Project. A) JPT, CHB, and CHS samples (*n* = 177) from the 1000G (the August 2010 release). B) JPT and CHB samples (*n* = 60) from the pilot 1 low coverage study data of 1000G (the March 2010 release). C) JPT samples (*n* = 90) from HapMap phase II+III (release #27). SNPs with minor allele frequency >0.05 were selected. (Step 3) Prediction of regulatory potential *in silico*. 3a) Regulatory potential (RP) scores were used for SNP selection, where an RP score >0.1 indicated the presence of regulatory elements. SNPs without RP scores were also selected. 3b) Prediction of regulatory elements by ChIP-seq data and DNase-seq data. (A) Transcription factor binding sites, (B) histone modification sites (CTCF binding, H3K4me1, H3K4me2, H3K4me3, H3K27ac, H3K9ac), and (C) DNase I hypersensitivity sites were evaluated. ChIP-seq and DNase-seq data derived from GM12878 EBV-transformed B cells were used for *NFKBIE* and *RTKN2*. DNase-seq data of Th1, Th2, and Jurkat cells were also used for *RTKN2*. (Step 4) Association data of the imputation-based GWAS using 1000G reference genotypes were used. SNPs with a significance level of *P*<0.05 were selected. SNPs without association data were also selected. (Step 5) EMSAs and luciferase assays were performed for evaluation of regulatory potentials *in vitro*.

To comprehensively search the two genomic regions for causal regulatory variants, we performed an integrated *in silico* and *in vitro* analysis with multiple steps (Figure 4 and Figures S3, S4). We first determined the target genomic region by selecting LD blocks containing disease-associated SNPs (*P*_GWAS_<1.0×10^−3^) (Step 1). We then extracted SNPs with frequencies of >0.05 from HapMap and 1000 Genome Project databases in the region (Step 2). We excluded uncommon variants (MAF<0.05) from the analysis because of their low imputation accuracy in the GWAS (93% of uncommon variants in *NFKBIE* and 76% in *RTKN2* exhibited *Rsq* <0.6). There is neither structural variation (>1 kbps) nor indels (100 bps to 1 kbs) that are common in the population (frequency >0.01) in these loci. To evaluate the *cis*-regulatory potential of sequences around the SNPs *in silico*, we used the regulatory potential (RP) score [21], [22]. This score was calculated based on the extent of sequence conservation among species or similarity with known regulatory motifs. We selected SNPs from the genomic elements with an RP score >0.1 (Step 3a). Subsequently, we selected SNPs from sites of transcriptional regulation as demonstrated by previous ChIP-seq studies (transcription factor binding sites [23], [24] and histone modification sites [25], [26]) or a DNase-seq study (DNase I hypersensitivity sites) [27] (Step 3b). Finally, these SNPs with regulatory potential were further screened out by the disease-association status (*P*<0.05) using an imputed GWAS dataset (Step 4). As a consequence, we selected 14 SNPs in *NFKBIE* and 10 SNPs in *RTKN2* that had regulatory potential predicted *in silico*.

**Figure 5:**
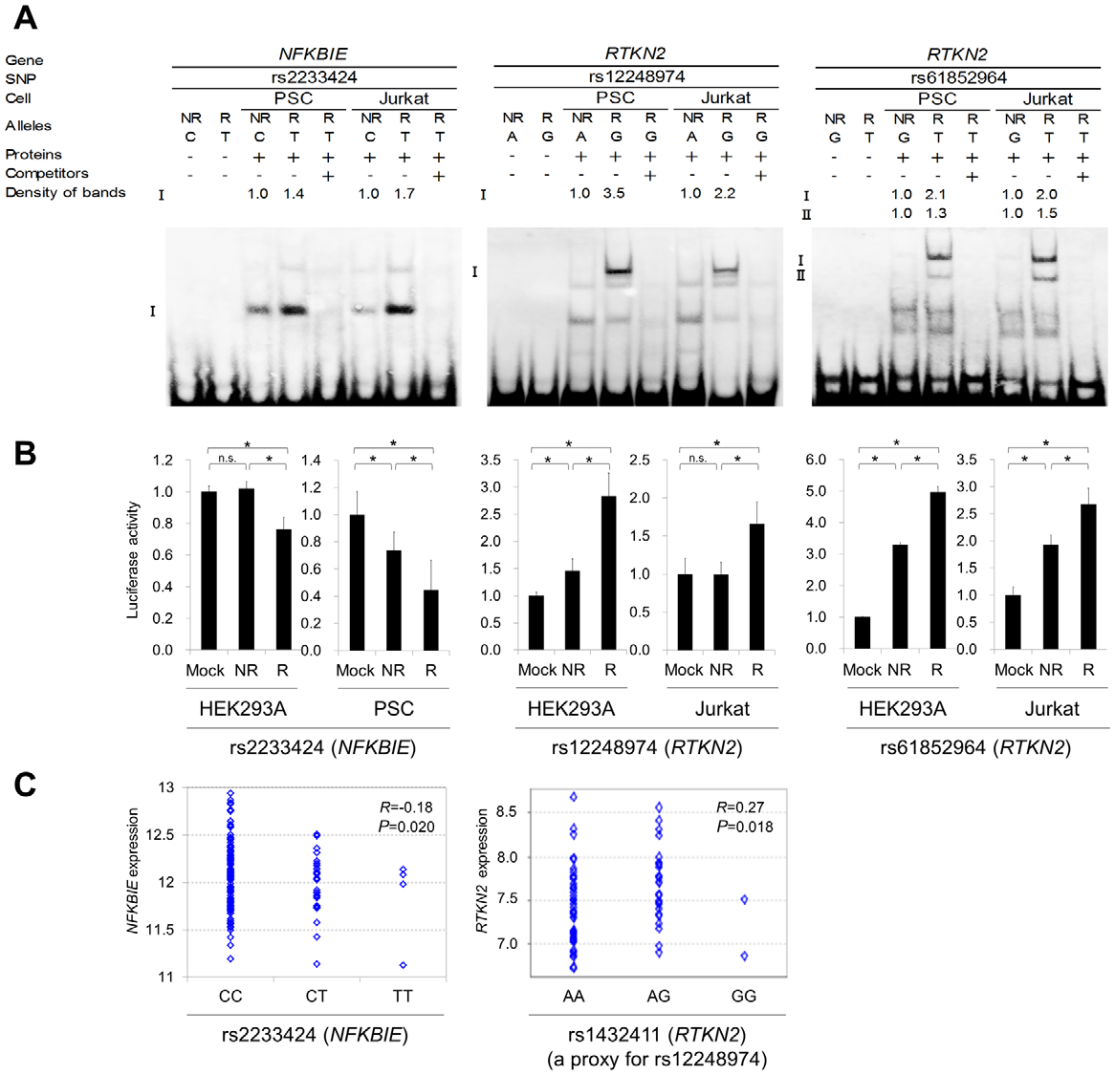
Evaluation of candidate regulatory SNPs *in vitro*. (A) Binding of nuclear factors from lymphoblastoid B-cells (PSC cells) and Jurkat cells to the 31-bp sequences around each SNP was evaluated by EMSA. Unlabeled probes in 200-fold excess as compared to the labeled probes were used for the competition experiment. The densities of the bands were quantified and normalized to that of the risk allele. rs2233424 in *NFKBIE* (C(NR)/T(R)) (left), rs12248974 (A(NR)/G(R)) (middle) and rs61852964 (G(NR)/T(R)) (right) in *RTKN2*. (B) Transcriptional activities were evaluated by luciferase assays. Each 31-bp oligonucleotide was inserted into the pGL4.24[*Luc2P*/minP] vector. Luc, luciferase; minP, minimal promoter. Transcfection was performed with HEK293A (for all the SNPs), PSC cells (for rs2233424), and Jurkat cells (for rs12248974 and rs61852964). rs2233424 (left), rs12248974 (middle), and rs61852964 (right). Data represent the mean ± s.d. Each experiment was performed in sextuplicate and independently repeated three times. *P<0.05 by Student's *t*-test. n.s.: not significant. (C) Liner regression analysis of the relationship between SNP genotype and gene expression level. *NFKBIE* expression data in lymphoblastoid B-cell lines of HapMap individuals (JPT+CHB, CEU and YRI; *n* = 151), and *RTKN2* expression data in primary T cells from umbilical cords of Western European individuals (*n* = 85) were used. The x-axis shows the SNP genotypes and the y-axis represents the log_2_-transformed gene expression level. R: correlation coefficient between SNP genotype and gene expression. Rs2233424 genotypes and *NFKBIE* expression level (left). The genotype classification by population: JPT+CHB, CC = 52, CT = 1; CEU, CC = 35, CT = 2; YRI, CC = 32, CT = 2, TT = 4. Rs1432411 genotypes and *RTKN2* expression level (right). Rs1432411 was used as a proxy SNP of rs12248974 (*r^2^* = 0.97).

To further investigate the regulatory potential of the SNPs, we evaluated 31-bp sequences around the SNPs by *in vitro* assays. First, we examined their ability to bind nuclear proteins by EMSAs (Step 5a) using nuclear extracts from lymphoblastoid B cells (PSC cells) and Jurkat cells. Of the 24 SNPs examined, nine SNPs displayed allelic differences, implying differential potential of transcriptional activity between these alleles (Figure 5A and Figure S5). We then evaluated the enhancing or repressing activity of the sequences by luciferase reporter assays (Step 5b). We cloned them into the pGL4.24 vector, which has minimal promoter activity, and transfected these constructs into HEK293A cells (for *NFKBIE* and *RTKN2*), lymphoblastoid B cells (for *NFKBIE*), and Jurkat cells (for *RTKN2*). Among the three SNPs examined in *NFKBIE*, the risk allele of rs2233424 (located −396 bps from the 5′ end) displayed stronger repression activity (Figure 2A and Figure 5B) than that of the non-risk allele. Among the six SNPs in *RTKN2*, the risk alleles of rs12248974 (approximately 10 kb from the 3′ end) and rs61852964 (−215 bps from the 5′ end) showed higher enhancing activity compared with the non-risk alleles (Figure 2A and Figure 5B). These results corresponded to the results of ASTQ analyses (Figure 3E, 3F). Other SNPs showed no allelic differences or had the opposite trend of transcriptional activity in the risk allele compared to the results of ASTQ analysis (Figure S6).

To confirm the regulatory potential of these SNPs, we investigated the correlation between genotypes and gene expression levels in lymphocytes utilizing the data from the previous eQTL studies. We evaluated the expression of *RTKN2* in primary T cells from Western European individuals by using Genevar software [28], [29]. Though *NFKBIE* is also expressed in primary T cells, the genotypes of rs2233424 are not available. We thus evaluated gene expression data of lymphoblastoid B-cell lines obtained from HapMap individuals (Japanese (JPT) + Han Chinese in Beijing (CHB), European (CEU), and African (YRI)) [30], [31] instead. The *NFKBIE* expression level decreased with the number of risk alleles of rs2233424 (*R* = −0.18, *P* = 0.020), and the *RTKN2* expression levels increased with that of rs1432411 (a proxy for rs12248974, *r^2^* = 0.97) (*R* = 0.27, *P* = 0.018) (Figure 5C), corresponding to the results of the *in vitro* assays. The data for rs61852964 in *RTKN2* was not available. Among the SNPs that displayed opposite transcriptional activities in the reporter assays compared to the results of ASTQ, the data for rs2233434, rs77986492, and rs3852694 (a proxy for rs1864836, *r^2^* = 1.0) were available (Figure S7 and S8). These SNPs displayed the opposite direction of the correlation trend as compared to the results of reporter assays, but parallel to ASTQ, implying that the regulatory effects observed in the *in vitro* assays were cancelled out by the effects of other regulatory variants on the same haplotype *in vivo*.

Finally, we validated the associations of these regulatory (r)SNPs observed in the imputed GWAS dataset. We directly genotyped them by TaqMan assay and confirmed significant associations (Table S8). As the candidate causal variants (nsSNPs and rSNPs) and the landmark SNPs of GWAS were in strong LD at each locus (Figure 2A, 2B), we evaluated the independent effect of each SNP by haplotype analysis in both loci (Table S9 and S10) and the conditional logistic regression analysis in *RTKN2* (Table S11). The conditional analysis was not performed in *NFKBIE* because three candidate causal variants were in strong LD (*r^2^*>0.9). However, the analyses for these two loci did not demonstrate any evidence of primary or independent effects across the candidate causal variants, and it remains a possibility that all of the functional variants were involved in the pathogenesis. In addition, although the landmark nsSNP (rs3125734) in *RTKN2* did not display any influence on NF-κB activity in our *in vitro* assays, rs3125734 might influence functions of *RTKN2* other than those in the NF-κB pathway; alternatively, it is still possible that rs3125734 tags the effects of other unknown variants, such as rare variants, in addition to the other two rSNPs (rs12248974 and rs61852964).

## Discussion

In the present study, we performed a replication study of our previously reported GWAS and identified variants in *NFKBIE* and *RTKN2* loci that were associated with RA susceptibility. The associations of *NFKBIE* and *RTKN2* loci have not been reported in other populations with genome-wide significance. However, rs2233434 in *NFKBIE* showed a suggestive association (589 cases vs. 1,472 controls, *P* = 0.0099, OR = 1.57, 95% CI = 1.11–2.21) in a previous meta-analysis in European populations [32]. The weak association signal in Europeans may be partially due to the lower frequency of the risk allele (0.04 in Europeans compared to 0.22 in Japanese). On the other hand, the association of rs3125734 in *RTKN2* was not observed in a GWAS meta-analysis of European populations (cases 5,539 vs. controls 20,169, *P* = 0.11, OR = 1.04, 95% CI = 0.99–1.09). As the association of *RTKN2* locus was also implicated in Graves' disease in a Han Chinese population [33], the association in *RTKN2* locus may be unique to Asian populations.

To find the disease causal variants in disease-associated loci, target re-sequencing and variant genotyping with a large sample set followed by conditional association analysis examining the independent effects of each variant would be the first step. For this purpose, a recent attempt to fine-map the known autoimmunity risk loci in Celiac disease (MIM 212750) using an “Immunochip” brought us several insights [34]. First, no stronger signals compared to the GWAS signals were detected in most of the known loci, while additional independent signals were found in several loci. Second, none of the genome-wide significant common SNP signals could be explained by any rare highly penetrant variants. Third, although the fine-mapping strategy could localize the association signals into finer scale regions, it could not identify the actual causal variants due to strong LD among the variants, indicating that an additional approach, such as functional evaluation of candidate variants, is needed.

In the present study, we focused on common variants to find causal variants. Instead of re-sequencing additional samples, we utilized the 1000 Genome Project dataset, where the theoretically estimated cover rate for common variants (frequency of >0.05) in our population is >0.99 [12], [35]. To fine-map the association signals, we performed imputation-based association analysis, where we could not find any association signals that statistically exceeded the effect of landmark SNPs (rs2233434 for *NFKBIE* and rs3125734 for *RTKN2*) in both gene regions (Figures S3 and S4). We also performed a conditional logistic regression analysis, and found no additional independent signals of association when conditioned on each landmark SNP (data not shown). Although the imputation-based association tests may yield some bias compared to direct genotyping of the variants, these results suggested that variants in strong LD with the landmark SNPs were strong candidates for causal variants.

Following the analysis of nsSNPs, we evaluated *cis*-regulatory effects of variants in the two regions by ASTQ analysis using both B-cell lines and primary cells (PBMC), the majority of which consisted of T and B lymphocytes. As the mechanism of gene-regulation is substantially different between cell types [26], ASTQ analysis in more specific cell types that are relevant to the disease etiology, such as Th1 and Th17 cells, would be ideal to evaluate the *cis*-regulatory effects of variants. In this context, a more comprehensive catalog of the eQTL database of multiple cell types should be established for genetic study of diseases. As our ASTQ analysis demonstrated *cis*-regulatory effects of variants in both regions, we then performed an integrated *in silico* and *in vitro* analysis to identify candidate regulatory variants. Accumulating evidence by recent ChIP-seq and DNase-seq studies suggested that *cis*-regulatory variants are located in the key regions of transcriptional regulation [26], [36], warranting the prioritization of variants before evaluation by *in vitro* assays. This could also minimize false-positive results of the *in vitro* assays. However, there may be additional causal variants, including rare variants, unsuccessfully selected at each step of our integrated screening. Therefore, the screening strategy should be refined as the quality and quantity of genomic databases improves in the future.

We identified multiple candidate causal variants in *NFKBIE* (two nsSNPs and one rSNP) and *RTKN2* (two rSNPs). We could not statistically distinguish the primary effect of each candidate causal variant, because these variants are in strong LD and on the same common haplotype. However, multiple causal variants could be involved in a single locus, which is also seen in another well-known autoimmune locus in 6q23 (*TNFAIP3* gene locus), where both an nsSNP and a regulatory variant have been shown to be functionally related to the disease [8], [37]. The risk haplotype of nsSNPs in *NFKBIE* (rs2233433 and rs2233434) showed an enhancement of NF-κB activity, which might reflect an impaired inhibitory effect of IκB-ε on nuclear translocation of NF-κB. On the other hand, down-regulated *NFKBIE* expression and up-regulated *RTKN2* expression were observed at the risk haplotypes, which may be regulated in *cis* by the rSNPs (rs2233424 in *NFKBIE*, rs12248974 and rs61852964 in *RTKN2*). As overexpression studies have also demonstrated dose-dependent attenuation of NF-κB activity by *NFKBIE*, and dose-dependent enhancement by *RTKN2*, the *cis*-regulatory effects of these rSNPs should enhance the NF-κB activity in the risk allele. Taken together with the effect of nsSNPs in *NFKBIE*, the enhancement of NF-κB activity may play a role in the pathogenesis of the disease. This is further supported by evidence that previous GWAS for RA have also identified genes related to the NF-κB pathway, such as *TNFAIP3* [13], v-rel reticuloendotheliosis viral oncogene homolog (*REL* [MIM 164910]) [5], TNF receptor-associated factor 1 (*TRAF1* [MIM 601711]) [3], and CD40 molecule TNF receptor superfamily member 5 (*CD40* [MIM 109535]) [38].

In conclusion, we identified *NFKBIE* and *RTKN2* as genetic risk factors for RA. Considering the allelic effect of both genes, enhanced NF-κB activity may play a role in the pathogenesis of the disease. Because NF-κB regulates the expression of numerous genes, including inflammatory and immune response mediators, NF-κB and its regulators identified by GWAS are promising targets for the treatment of RA.

## Materials and Methods

### Ethics statement

All subjects were of Japanese origin and provided written informed consent for participation in the study, which was approved by the ethical committees of the institutional review boards.

### Subjects

A total of 7,907 RA cases, 657 SLE cases, 1,783 GD cases, and 35,362 control subjects were enrolled in the study through medical institutes in Japan under the support of the BioBank Japan Project, Center for Genomic Medicine at RIKEN, the University of Tokyo, Tokyo Women's Medical University, and Kyoto University. The same case and control samples were used in the previous meta-analysis of GWASs in the Japanese population (Table S1) [15] . RA and SLE subjects met the revised American College of Rheumatology (ACR) criteria for RA [39]. Diagnosis of individuals with GD was established on the basis of clinical findings and results of the routine examinations for circulating thyroid hormone and thyroid-stimulating hormone concentrations, thyroid-stimulating hormone receptors, ultrasonography, ^[99m]^TCO_4_^−^ (or [^123^I]) uptake, and thyroid scintigraphy. DNAs were extracted from peripheral blood cells using a standard protocol. Total RNAs were also extracted from PBMCs of healthy individuals (*n* = 20) using an RNeasy kit (QIAGEN, Valencia, CA, USA). Details of the samples are summarized in Table S1.

### Genotyping and quality control

In the GWAS, RA cases and controls were genotyped using Illumina Human610-Quad and Illumina Human 550v3 Genotyping BeadsChips (Illumina, San Diego, CA, USA), respectively, and quality control of genotyping was performed as described previously [6]. For replication study of candidate loci, a landmark SNP was selected from each locus that satisfied 5×10^−8^<*P_GWAS_*<5×10^−5^ in the GWAS. If multiple candidate SNPs existed within ±100 kb, the SNP with the lowest *P*-value was selected. All case subjects in the replication study and both case and control subjects in the validation study of candidate causal variants were genotyped using TaqMan SNP genotyping assays (Table S12) (Applied Biosystems, Foster City, CA, USA) with an ABI Prism 7900HT Sequence Detection System (Applied Biosystems). Because of the availability of DNA samples, only a part of the control subjects were genotyped for the validation study (*n* = 3,290, 97.3%). To enlarge the number of subjects and enhance statistical power for replication studies, we used genotype data obtained from other GWAS projects genotyped using the Illumina platforms for the replication control panels (Table S1). All SNPs were successfully genotyped with call rates >0.98 and were in Hardy-Weinberg equilibrium (HWE) in control subjects (*P*>0.05 as examined by χ^2^ test), except for rs2233434, which displayed a deviation from HWE (*P* = 0.00091). To evaluate possible genotyping biases between the platforms, we also genotyped rs2233434 and rs3125734 by TaqMan assays for randomly selected subjects genotyped using other genotyping platforms (*n* = 376), yielding high concordance rates of ≥0.99.

### Association analysis

The associations of the SNPs were tested with the Cochran-Armitage trend test. Combined analysis was performed with the Mantel-Haenszel method. Haplotype association analysis and haplotype-based conditional association analysis were performed using Haploview v4.2 and the PLINK v1.07 program (see URLs) [40], respectively. The SNPs that were not genotyped in the GWAS were imputed using MACH 1.0.16 (see URLs), with genotype data from the 1000 Genome Project (JPT, CHB, and Han Chinese South (CHS): 177 individuals) as references (August 2010 release) [41]. All the imputed SNPs demonstrated *Rsq* values more than 0.60.

### DNA re-sequencing

Unknown variants in the coding sequences of *NFKBIE* and *RTKN2* were revealed by directly sequencing the DNA of 48 individuals affected with RA. DNA fragments were amplified with the appropriate primers (Table S13). Purification of PCR products was performed with Exonuclease I (New England Biolabs, Ipswich, MA, USA) and shrimp alkaline phosphatase (Promega, Madison, WI, USA). The amplified DNAs were sequenced using the BigDye Terminator v3.1 Cycle Sequencing kit (Applied Biosystems), and signals were detected using an ABI 3700 DNA Analyzer (Applied Biosystems).

### Construction of haplotype-specific expression vectors

The full coding regions were amplified using cDNAs prepared from an Epstein-Barr virus-transfected lymphoblastoid B-cell line (Pharma SNP Consortium (PSC), Osaka, Japan) for *NFKBIE* (NM_004556.2) and from Jurkat cells (American Type Culture Collection (ATCC), Rockville, MD, USA) for *RTKN2* (NM_145307.2) with appropriate primers (Table S14) and DNA polymerases. PCR products were inserted into the pcDNA3.1D/V5-His-TOPO vector (Invitrogen, Camarillo, CA, USA) using the TaKaRa Ligation kit ver. 2.1 (Takara Bio Inc, Shiga, Japan), and mutagenized using the AMAP Multi Site-Directed Mutagenesis Kit (MBL, Nagoya, Japan). Each construct was then transformed into Jet Competent *Escherichia coli* cells (DH5α) (BioDynamics Laboratory Inc., Tokyo, Japan). These plasmids were purified using an Endofree Plasmid Maxi Kit (QIAGEN) after confirmation of the sequence.

### NF-κB reporter assay

Human embryonic kidney (HEK) 293A cells (Invitrogen) were cultured in Dulbecco's modified Eagle's medium (Sigma-Aldrich, St. Louis, MO, USA) supplemented with 10% fetal bovine serum (BioWest, Nuaillé, France), 1% penicillin/streptomycin (Invitrogen), and 0.1 mM MEM Non-Essential Amino Acids (Invitrogen). Various doses of the haplotype-specific expression vector (0.0025–0.02 µg for *NFKBIE* and 0.1–0.8 µg for *RTKN2*), pGL4.32[*luc2P*/NF-κB-RE/Hygro] vector (Promega) (0.05 µg and 0.0125 µg, respectively), and pRL-TK vector (an internal control for transfection efficiency) (0.45 µg and 0.15 µg, respectively) were transfected into the HEK293A cells using the Lipofectamine LTX transfection reagent (Invitrogen) according to the manufacturer's protocol. The total amounts of DNAs were adjusted with empty pcDNA3.1 vector. After 22 h, cells were incubated with 1 ng/ml TNF-α (Sigma) for 2 h or with medium alone. Cells were collected, and luciferase activity was measured using a Dual-Luciferase Reporter Assay system (Promega) and a GloMax-Multi+ Detection System (Promega). Each experiment was independently repeated three times, and sextuplicate samples were assayed each time.

### Western blotting

After 24 h of transfection as described for the NF-κB reporter assay, cells were lysed in NP-40 lysis buffer (150 mM NaCl, 1% NP-40, 50 mM Tris-HCl at pH 8.0, and a protease inhibitor cocktail), and incubated on ice for 30 min. After centrifugation, the supernatant fraction was collected and 4×Sodium dodecyl sulfate (SDS) sample buffer was added. After denaturation at 95°C for 5 min, proteins were analyzed by SDS-polyachrylamide gel electrophoresis (PAGE) on a 5% to 20% gradient gel (Wako, Osaka, Japan) and were transferred to polyvinylidene difluouride (PVDF) membranes (Millipore, Billerica, MA, USA). Target proteins on the membrane were probed with antibodies (mouse anti-V5 tag (Invitrogen), anti-β-actin-HRP (an internal control), and goat anti-mouse IgG2a-HRP (Santa Cruz Biotechnology, Santa Cruz, CA, USA)), visualized using enhanced chemiluminescence (ECL) detection reagent (GE Healthcare, Pollards Wood, UK), and detected using a LAS-3000 mini lumino-image analyzer (Fujifilm, Tokyo, Japan). Band intensities were measured using MultiGauge software (Fujifilm).

### Allele-specific transcript quantification (ASTQ) analysis

ASTQ analysis was performed as previously described [42]. Total RNAs and genomic DNAs were extracted from PBMCs and lymphoblastoid B-cell lines. cDNAs were synthesized using TaqMan reverse transcription reagents (Applied Biosystems). We selected SNPs (rs2233434 (A/G) for *NFKBIE* and rs3125734 (C/T) for *RTKN2*) as target SNPs. Allele-specific gene expression was measured by TaqMan SNP genotyping probes for these SNPs (Applied Biosystems). To make a standard curve, we selected two individuals that had homozygous genotypes of each target SNP. We mixed these DNAs at nine different ratios and detected the intensities. The log_2_ of (risk allele/non-risk allele intensity) for each SNP was plotted against the log_2_ of mixing homozygous DNAs. We generated a standard curve (linear regression line; y = ax+b), where y is the log_2_ of (risk allele/non-risk allele intensity) at a given mixing ratio, x is the log_2_ of the mixing ratio, a is the slope, and b is the intercept. We then measured the allelic ratio for each cDNA and genomic DNA from each individual by real-time TaqMan PCR. Based on a standard curve, we calculated the allelic ratio of cDNAs and genomic DNAs. Intensities were detected using an ABI Prism 7900HT Sequence Detection System (Applied Biosystems).

### Electrophoretic mobility shift assays (EMSA)

EMSA and preparation of nuclear extract from lymphoblastoid B-cell lines and Jurkat cells were performed as previously described [43]. Cells were cultured in RPMI-1640 medium (Sigma-Aldrich) supplemented with 10% fetal bovine serum and 1% penicillin/streptomycin. Following stimulation with 50 ng/ml phorbol myristate acetate (Sigma-Aldrich) for 2 h, cells were collected and suspended in buffer A (20 mM HEPES at pH 7.6, 20% glycerol, 10 mM NaCl, 1.5 mM MgCl_2_, 0.2 mM EDTA at pH 8.0, 1 mM DTT, 0.1% NP-40, and a protease inhibitor cocktail) for 10 min on ice. After centrifugation, the pellets were resuspended in buffer B (which contains buffer A with 500 mM NaCl). Following incubation on ice for 30 min and centrifugation to remove cellular debris, the supernatant fraction containing nuclear proteins was collected. Oligonucleotides (31-bp) were designed that corresponded to genomic sequences surrounding the SNPs (Table S15). Single-stranded oligonucleotide probes were labeled using a Biotin 3′ End DNA Labeling Kit (Pierce Biotechnology, Rockford, IL, USA), and sense and antisense oligonucleotides were then annealed. DNA-protein interactions were detected using a LightShift Chemiluminescent EMSA kit (Pierce Biotechnology). The DNA-protein complexes were separated on a non-denaturing 5% polyachrylamide gel in 1×TBE (Tris-borate-EDTA) running buffer for 60 min at 150 V. The DNA-protein complexes were then transferred from the gel onto a nitrocellulose membrane (Ambion, Carlsbad, CA, USA), and were cross-linked to the membrane by exposure to UV light. Signals were detected using a LAS-3000 mini lumino-image analyzer (Fujifilm). Allelic differences were analyzed using MultiGauge software (Fujifilm) by measuring the intensity of the bands.

### Luciferase assay

Oligonucleotides (31-bp) were designed as described for the EMSAs (Table S15), and complementary sense and antisense oligonucleotides were annealed. To construct luciferase reporter plasmids, pGL4.24[*luc2P*/minP] vector (Promega) was digested with restriction enzymes (XhoI and BglII) (Takara Bio Inc), and annealed oligonucleotide was ligated into a pGL4.24 vector upstream of the minimal promoter. HEK293A (*n* = 2.5×10^5^), lymphoblastoid B-cell lines (*n* = 2.0×10^6^) and Jurkat (*n* = 5.0×10^5^) cells were transfected with the allele-specific constructs (0.4 µg, 1.8 µg and 2.5 µg, respectively) and the pRL-TK vector (0.1 µg, 0.2 µg and 0.25 µg, respectively) using the Lipofectamine LTX transfection reagent (for HEK293A and Jurkat cells) and Amaxa nucleofector kit (Lonza, Basel, Switzerland) (for lymphoblastoid B-cell lines). Cells were collected, and luciferase activity was measured as described for the NF-κB reporter assay. Each experiment was independently repeated three times and sextuplicate samples were assayed each time.

### Correlation analysis between gene expression and genotypes

The expression data in lymphoblastoid B-cell lines derived from HapMap individuals (*n* = 210; JPT, CHB, CEU, and YRI) and in primary T cells from umbilical cords of Western European individuals (*n* = 85) from the database of the Gene Expression Variation (Genevar) project were used. SNP genotypes were obtained from HapMap and 1000 Genome Project databases. The expression levels were regressed with the genotype in a liner model. The statistical significance of regression coefficients was tested using Student's *t*-test.

### Statistical analysis

We used χ^2^ contingency table tests to evaluate the significance of differences in allele frequency in the case-control subjects. We defined haplotype blocks using the solid spine of LD definition of Haploview v4.2, and estimated haplotype frequency and calculated pairwise LD indices (*r^2^*) between pairs of polymorphisms using the Haploview program. Luciferase assay data and ASTQ analysis data were analyzed by Student's *t*-test.

### Web resources

The URLs for data presented herein are as follows:

PLINK, http://pngu.mgh.harvard.edu/~purcekk/plink

MACH, http://www.sph.umich.edu/csg/abecasis/mach/

UCSC Genome Browser, http://genome.ucsc.edu/;

Genevar, http://www.sanger.ac.uk/resources/software/genevar/

HapMap Project, http://www.HapMap.org/

1000 Genome Project, http://www.1000genomes.org

Online Mendelian Inheritance in Man (OMIM), http://www.omim.org/

## Supporting Information

Figure S1 NF-kB activity was influenced by nsSNPs in *NFKBIE*. NF-κB activities were evaluated by luciferase assays. Allele specific construct, pGL4.32[*luc2P*/NF-κB-RE] luciferase vector, and pRL-TK vector were transfected into HEK293A cells. Four haplotypes (rs2233434-rs2233433; A-C, G-C, A-T, and G-T) were examined. (rs2233434: A = non-risk (NR), G = risk (R); rs2233433: C = NR, T = R). Twenty-two hours after transfection, cells were stimulated with medium alone (A) or TNF-α (B) for 2 h. Data represent the mean ± s.d. Each experiment was performed in sextuplicate, and experiments were independently repeated three times. *P<0.05 and **P<1.0×10^−5^ by Student's *t*-test. n.s.: not significant.
(TIF)

Figure S2 Allelic imbalance of expression in *NFKBIE*. ASTQ was performed using samples from individuals heterozygous for rs2233434 (G/A) in *NFKBIE*. Genomic DNAs and cDNAs were extracted from lymphoblastoid B cells (*n* = 9). The y-axis shows the log_2_ ratio of the transcript amounts in target SNPs (risk allele/non-risk allele). The top bar of the box-plot represents the maximum value and the lower bar represents the minimum value. The top of box is the third quartile, the bottom of box is the first quartile, and the middle bar is the median value. The circle is an outlier. *P = 5.3×10^−4^ by Student's *t*-test.
(TIF)

Figure S3 SNP selection using *in silico* analysis in the *NFKBIE* region. Step 1: Definition of the target region. *P-*values of the SNPs in the GWAS (top) and genomic structure (middle), and the *D′*-based LD map (bottom). The green diamond shapes represent the -log_10_ of the Cochran-Armitage trend *P-*values. The dashed line indicates the significance threshold (*P*<1×10^−3^). The LD map was drawn based on genotype data of the 1000 Genome Project (JPT, CHB and CHS: 177 samples) using Haploview software v4.2. LD blocks were defined by the solid spine method. The red box (top) represents the target region of the *in silico* analysis (Chr6: 44,336,140-44,394,125). Step 2: Target SNPs were extracted from public databases (HapMap and 1000 Genome Project). SNPs with MAF >0.05 were selected. Step 3: Evaluation of regulatory potential. Step 3a: The regulatory potential (RP) score was calculated for sequences surrounding the SNPs by ESPERR (evolutionary and sequence pattern extraction through reduced representations) method. SNPs with RP score >0.1 were selected. Step 3b: Subsequently, SNPs within the predicted, regulatory genomic elements were selected by using ChIP-seq data of transcription factor binding sites (Txn factor), histone modification sites (CTCF binding, H3K4me1, H3K4me2, H3K4me3, H3K27ac, H3K9ac) or DNase-seq data of DNase I hypersensitivity sites (DNase HS). ChIP-seq data and DNase-seq data used the signals derived from GM12878 EBV-transformed B cells. All these analyses of Steps 2 to 3 were performed by using the UCSC genome browser. Step 4: Evaluation of disease association. Association data of both genotyped (green diamonds) and imputed (black diamonds) SNPs in the GWAS samples were used. Red triangles represent 14 extracted SNPs *in silico*. The dashed line indicates the significance threshold (*P*<0.05).
(TIF)

Figure S4 SNP selection using *in silico* analysis in the *RTKN2* region. SNP selection in the *RTKN2* region was performed the same as in the case of the *NFKBIE* region as described in Figure S3, except that we used DNase-seq data derived from Th1, Th2, and Jurkat cells in addition to GM12878 EBV-transformed B cells.
(TIF)

Figure S5 Results of EMSAs for candidate regulatory SNPs. Binding affinities of nuclear factors from lymphoblastoid B-cells (PSC cells) and Jurkat cells to the 31-bp sequences around each allele of the candidate regulatory SNPs were evaluated by EMSA. Nuclear factors from PSC cells were used for *NFKBIE*, and Jurkat cells were used for *RTKN2*. 14 SNPs in *NFKBIE* (A) and 10 SNPs in *RTKN2* (B) were tested. NR: non-risk allele; R: risk allele. Arrows indicate bands showing allelic differences in each SNP.
(TIF)

Figure S6 Luciferase assays for regulatory SNPs. Transcriptional activities of the 31-bp genomic sequences around the SNPs were evaluated by luciferase assays. Each oligonucleotide was inserted into the pGL4.24[*luc2P*/minP] vector upstream of the minimal promoter (minP), and allele-specific constructs were transfected into HEK293A cells. Relative luciferase activity is expressed as the ratio of luciferase activity of each allele-specific construct to the luciferase activity of the mock construct. Data represent the mean ± s.d. Each experiment was independently repeated three times, and each sample was measured in sextuplicate. *P<1×10^−3^ by Student's *t*-test. n.s.: not significant. (A) rs2233434 and rs77986492 in the *NFKBIE* region. (B) rs3864793, rs1864836, rs4979765, and rs4979766 in the *RTKN2* region. NR: non-risk allele; R: risk allele.
(TIF)

Figure S7 The correlation between *NFKBIE* expression and rs2233434 and rs77986492 genotypes. Linear regression analysis of the relationship between SNP genotypes and *NFKBIE* expression. Gene expression data from EBV-transformed lymphoblastoid B cell lines of HapMap individuals (JPT+CHB, CEU, and YRI). (A) rs2233434 (*n* = 204) and (B) rs77986492 (*n* = 152). The genotype classification by population: rs2233434 (JPT+CHB, AA = 61, AG = 28, GG = 1; CEU, AA = 52, AG = 2; YRI, AA = 53, AG = 72) and rs77986492 (JPT+CHB, CC = 52, CT = 24; CEU, CC = 35, CT = 2; YRI, CC = 38, CT = 1). The x-axis shows SNP genotypes and the y-axis represents the log_2_-transformed *NFKBIE* expression level. *R*: the correlation coefficient between *NFKBIE* expression and SNP genotype.
(TIF)

Figure S8 The correlation between *RTKN2* expression and rs3852694 genotypes. Linear regression analysis of the relationship between the rs3852694 genotype and *RTKN2* expression. Rs3852694 was used as a proxy SNP of rs1864836 (*r^2^* = 1.0). Gene expression data in primary T cells from umbilical cords of Western European individuals (*n* = 85) were presented by using Genevar software. The x-axis shows the rs3852694 genotypes (AA, AG, GG) and the y-axis represents the log_2_-transformed *RTKN2* expression level. *R*: the correlation coefficient between *RTKN2* expression and rs3852694 genotype.
(TIF)

Table S1 Summary of samples.
(DOC)

Table S2 Association results of the GWAS and 1st replication study.
(DOC)

Table S3 Association analysis of *NFKBIE* and *RTKN2* with autoimmune diseases.
(DOC)

Table S4 Association analysis of nsSNPs with RA.
(DOC)

Table S5 Haplotype association study of nsSNPs in *NFKBIE*.
(DOC)

Table S6 Haplotype association study of nsSNPs in *RTKN2*.
(DOC)

Table S7 Predicting the effects of nsSNPs on protein function.
(DOC)

Table S8 Association analysis of candidate rSNPs with RA.
(DOC)

Table S9 Haplotype association study of candidate causal SNPs in *NFKBIE*.
(DOC)

Table S10 Haplotype association study of candidate causal SNPs in *RTKN2*.
(DOC)

Table S11 The conditional haplotype-based association analysis of candidate causal SNPs in *RTKN2*.
(DOC)

Table S12 Probes and Primers used for TaqMan assays.
(DOC)

Table S13 Primers used for DNA re-sequencing.
(DOC)

Table S14 Primers used for construction of expression vectors.
(DOC)

Table S15 Oligonucleotides used for EMSAs and Luciferase assays.
(DOC)
