# Supplementary figures and images for: Functional Variants in NFKBIE and RTKN2 Involved in Activation of the NF-κB Pathway Are Associated with Rheumatoid Arthritis in Japanese
Source: PLoS Genet. 2012 Sep 13;8(9):e1002949. doi: 10.1371/journal.pgen.1002949 (PMC3441678; doi:10.1371/journal.pgen.1002949)

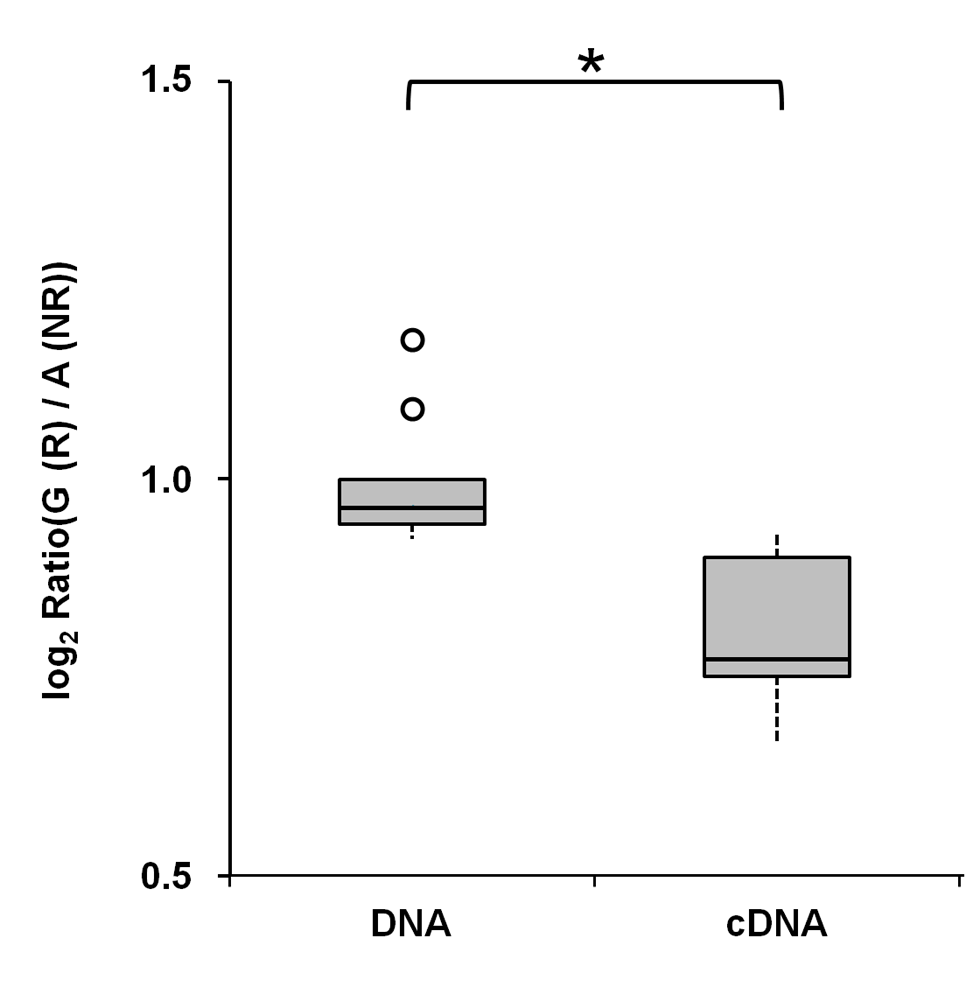

Supplement: Figure S2 — Allelic imbalance of expression in NFKBIE. ASTQ was performed using samples from individuals heterozygous for rs2233434 (G/A) in NFKBIE. Genomic DNAs and cDNAs were extracted from lymphoblastoid B cells (n = 9). The y-axis shows the log2 ratio of the transcript amounts in target SNPs (risk allele/non-risk allele). The top bar of the box-plot represents the maximum value and the lower bar represents the minimum value. The top of box is the third quartile, the bottom of box is the first quartile, and the middle bar is the median value. The circle is an outlier. *P = 5.3×10^−4^ by Student's t-test. (TIF) [file pgen.1002949.s002.tif]

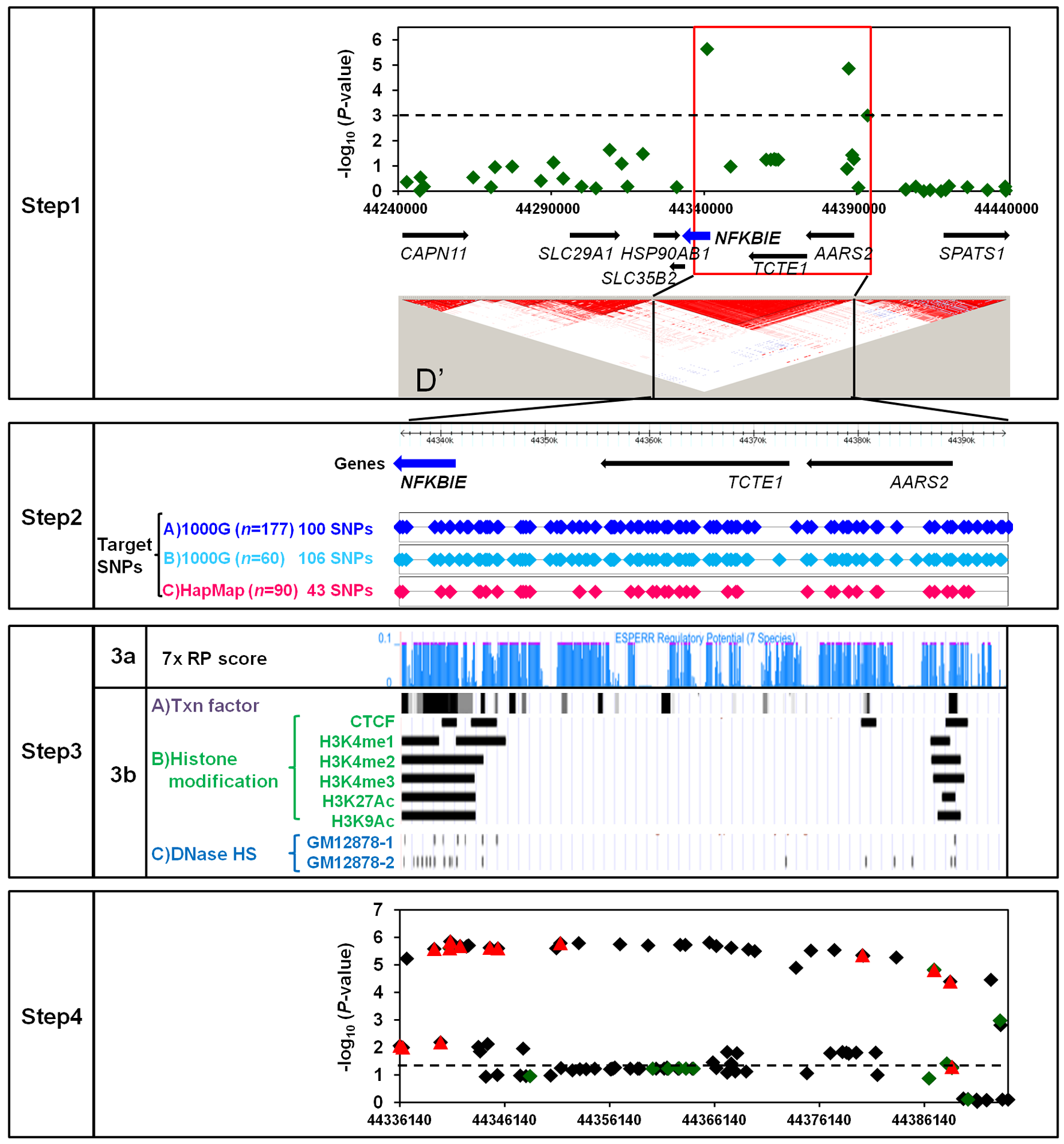

Supplement: Figure S3 — SNP selection using in silico analysis in the NFKBIE region. Step 1: Definition of the target region. P-values of the SNPs in the GWAS (top) and genomic structure (middle), and the D′-based LD map (bottom). The green diamond shapes represent the -log10 of the Cochran-Armitage trend P-values. The dashed line indicates the significance threshold (P<1×10^−3^). The LD map was drawn based on genotype data of the 1000 Genome Project (JPT, CHB and CHS: 177 samples) using Haploview software v4.2. LD blocks were defined by the solid spine method. The red box (top) represents the target region of the in silico analysis (Chr6: 44,336,140-44,394,125). Step 2: Target SNPs were extracted from public databases (HapMap and 1000 Genome Project). SNPs with MAF >0.05 were selected. Step 3: Evaluation of regulatory potential. Step 3a: The regulatory potential (RP) score was calculated for sequences surrounding the SNPs by ESPERR (evolutionary and sequence pattern extraction through reduced representations) method. SNPs with RP score >0.1 were selected. Step 3b: Subsequently, SNPs within the predicted, regulatory genomic elements were selected by using ChIP-seq data of transcription factor binding sites (Txn factor), histone modification sites (CTCF binding, H3K4me1, H3K4me2, H3K4me3, H3K27ac, H3K9ac) or DNase-seq data of DNase I hypersensitivity sites (DNase HS). ChIP-seq data and DNase-seq data used the signals derived from GM12878 EBV-transformed B cells. All these analyses of Steps 2 to 3 were performed by using the UCSC genome browser. Step 4: Evaluation of disease association. Association data of both genotyped (green diamonds) and imputed (black diamonds) SNPs in the GWAS samples were used. Red triangles represent 14 extracted SNPs in silico. The dashed line indicates the significance threshold (P<0.05). (TIF) [file pgen.1002949.s003.tif]

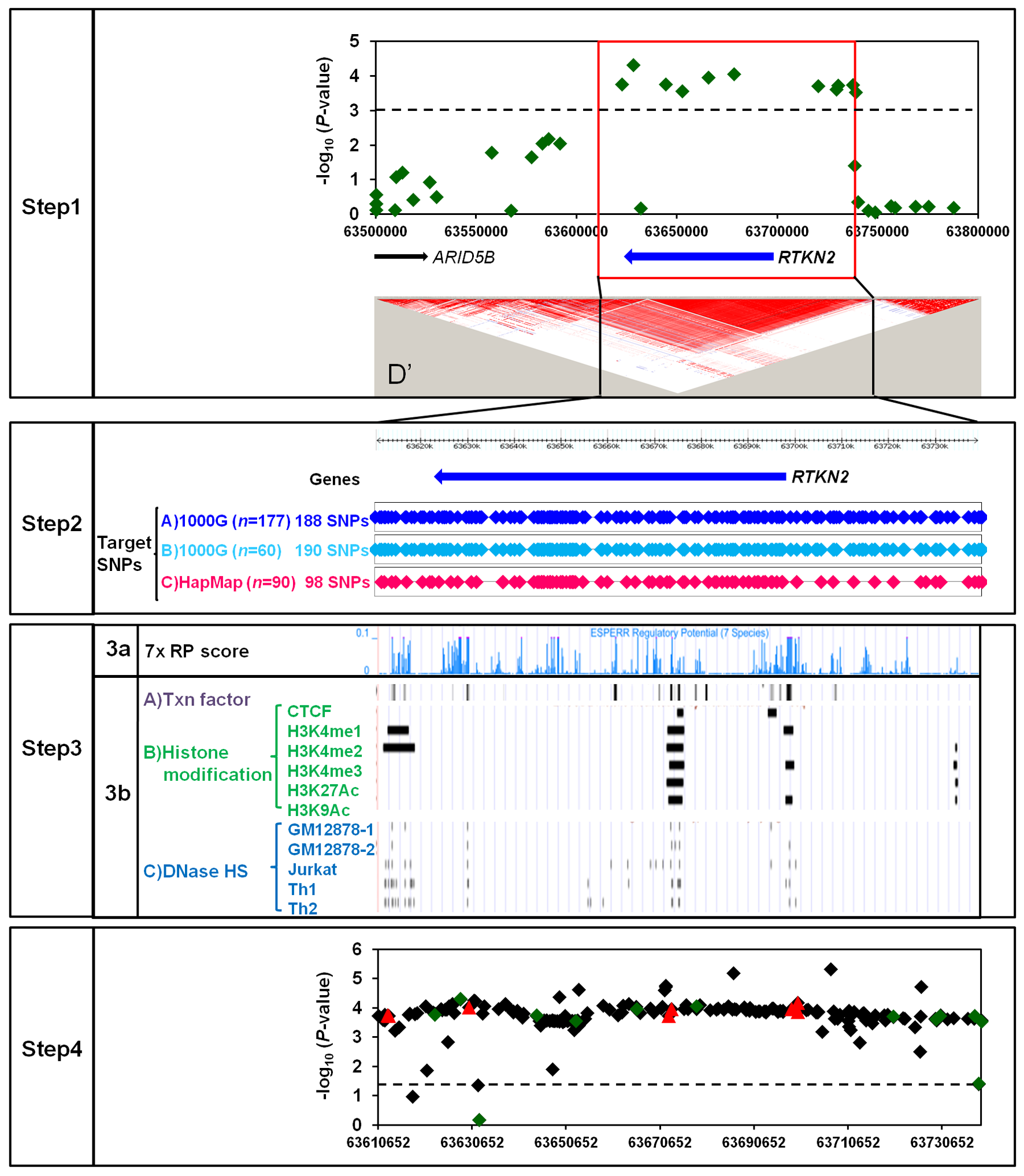

Supplement: Figure S4 — SNP selection using in silico analysis in the RTKN2 region. SNP selection in the RTKN2 region was performed the same as in the case of the NFKBIE region as described in Figure S3, except that we used DNase-seq data derived from Th1, Th2, and Jurkat cells in addition to GM12878 EBV-transformed B cells. (TIF) [file pgen.1002949.s004.tif]

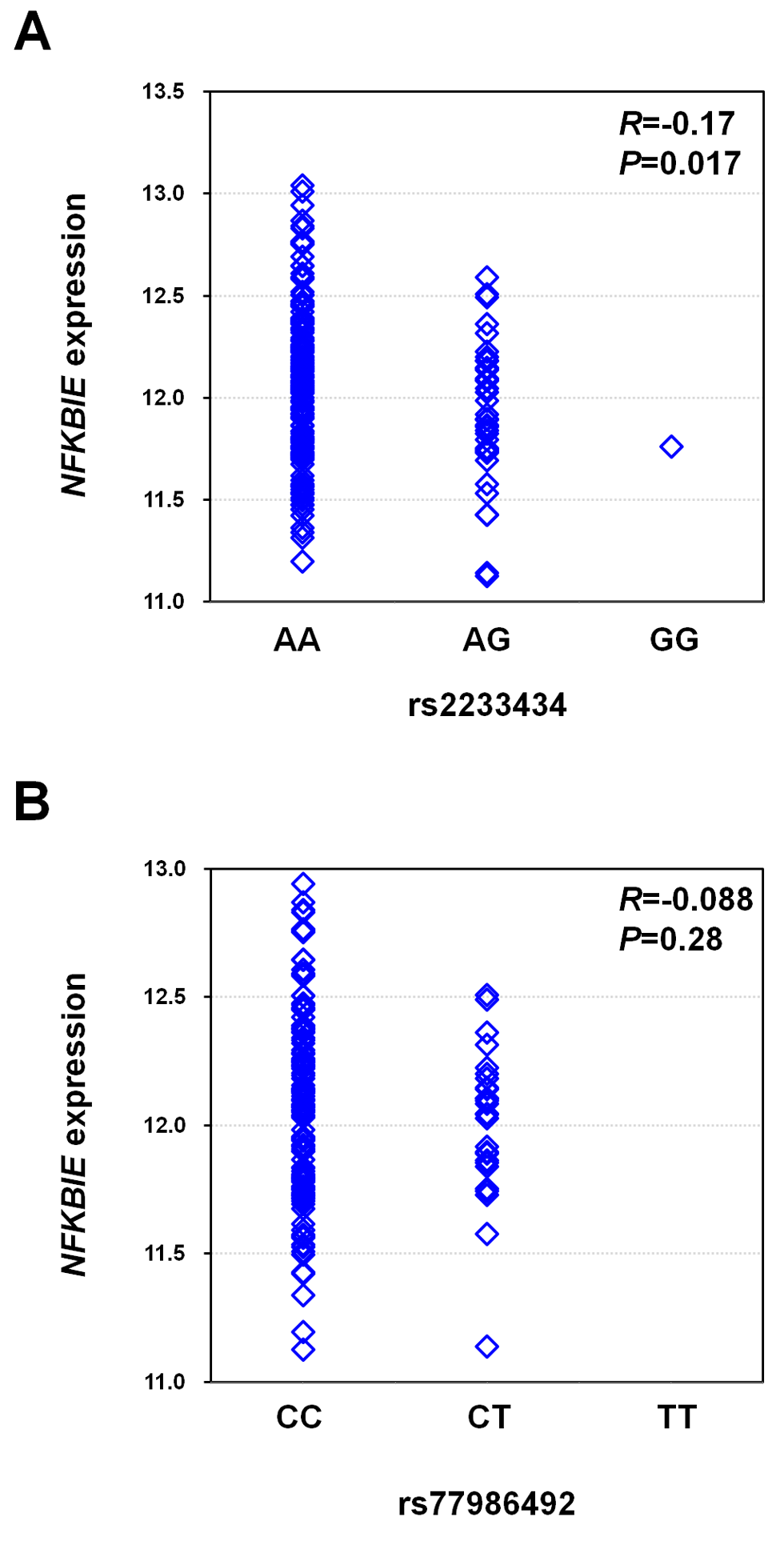

Supplement: Figure S7 — The correlation between NFKBIE expression and rs2233434 and rs77986492 genotypes. Linear regression analysis of the relationship between SNP genotypes and NFKBIE expression. Gene expression data from EBV-transformed lymphoblastoid B cell lines of HapMap individuals (JPT+CHB, CEU, and YRI). (A) rs2233434 (n = 204) and (B) rs77986492 (n = 152). The genotype classification by population: rs2233434 (JPT+CHB, AA = 61, AG = 28, GG = 1; CEU, AA = 52, AG = 2; YRI, AA = 53, AG = 72) and rs77986492 (JPT+CHB, CC = 52, CT = 24; CEU, CC = 35, CT = 2; YRI, CC = 38, CT = 1). The x-axis shows SNP genotypes and the y-axis represents the log2-transformed NFKBIE expression level. R: the correlation coefficient between NFKBIE expression and SNP genotype. (TIF) [file pgen.1002949.s007.tif]

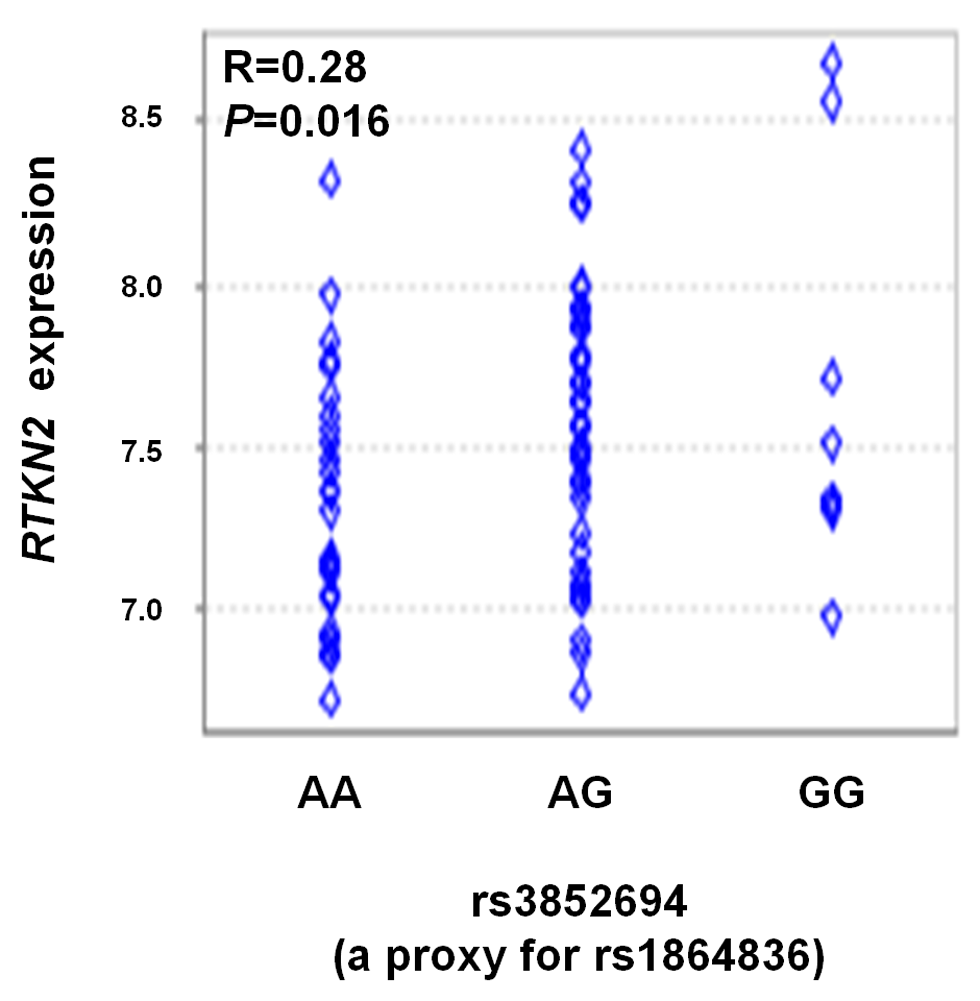

Supplement: Figure S8 — The correlation between RTKN2 expression and rs3852694 genotypes. Linear regression analysis of the relationship between the rs3852694 genotype and RTKN2 expression. Rs3852694 was used as a proxy SNP of rs1864836 (r2 = 1.0). Gene expression data in primary T cells from umbilical cords of Western European individuals (n = 85) were presented by using Genevar software. The x-axis shows the rs3852694 genotypes (AA, AG, GG) and the y-axis represents the log2-transformed RTKN2 expression level. R: the correlation coefficient between RTKN2 expression and rs3852694 genotype. (TIF) [file pgen.1002949.s008.tif]
