# Supplementary material for: Functional Variants in NFKBIE and RTKN2 Involved in Activation of the NF-κB Pathway Are Associated with Rheumatoid Arthritis in Japanese
Source: PLoS Genet. 2012 Sep 13;8(9):e1002949. doi: 10.1371/journal.pgen.1002949 (PMC3441678; doi:10.1371/journal.pgen.1002949)
Supplement: Table S1 — Summary of samples. (DOC) [file pgen.1002949.s009.doc]

**Table S1. Summary of samples.**

| Disease | Study stages  in the present study | Source^b^ | Genotyping platform^c^ | No. of samples | Study stages  in the previous meta-analysis of GWASs^d^ |
| --- | --- | --- | --- | --- | --- |
| Rheumatoid arthritis | GWAS^a^ | BBJP | Illumina HumanHap610-Quad | 2,303 | GWAS |
|  | Replication study-1 | BBJP and RIKEN | Taqman genotyping system | 2,187 | Replication study-1 |
|  | Replication study-2 | IORRA and Kyoto university | Taqman genotyping system | 3,417 | GWAS |
|  |  |  |  |  | Replication study-2 |
|  |  |  |  |  |  |
| Control | GWAS | BBJP | Illumina HumanHap550 | 3,380 | GWAS |
|  |  |  |  |  | Replication study-1 |
|  | Replication study-1 | BBJP | Illumina HumanHap610-Quad | 28,219 | GWAS |
|  |  |  |  |  | Replication study-1 |
|  | Replication study-2 | Kyoto university | Illumina HumanHap550 | 3,763 | Replication study-2 |
|  |  |  | Illumina HumanHap610-Quad |  |  |
|  |  |  |  |  |  |
| Systemic lupus erythematosus | - | RIKEN | Taqman genotyping system | 657 | - |
| Graves’ disease | - | BBJP | Taqman genotyping system | 1,783 | - |

a: GWAS, Genome-wide association study

b: BBJP, BioBank Japan project; IORRA, the Institute of Rheumatology Arthritis

c: Infinium I and II assays were used in Illumina beadchips.

d: The meta-analysis of GWASs for RA in the Japanese population performed by Y. Okada et al (*Nat Genet* 2012).
