# Supplementary material for: Functional Variants in NFKBIE and RTKN2 Involved in Activation of the NF-κB Pathway Are Associated with Rheumatoid Arthritis in Japanese
Source: PLoS Genet. 2012 Sep 13;8(9):e1002949. doi: 10.1371/journal.pgen.1002949 (PMC3441678; doi:10.1371/journal.pgen.1002949)
Supplement: Table S2 — Association results of the GWAS and 1st replication study. (DOC) [file pgen.1002949.s010.doc]

**Table S2. Association results of the GWAS and 1st replication study.**

|  |  |  |  |  | GWAS | | | | Replication study-1 | | | | Combined analysis | |
| --- | --- | --- | --- | --- | --- | --- | --- | --- | --- | --- | --- | --- | --- | --- |
|  |  |  |  | Allele | Allele 1 Frequency | |  |  | Allele 1 Frequency | |  |  |  | |
| Rank | dbSNP ID | Chr | Gene | (1/2) | Case | Control | *P*_GWAS_^a^ | OR (95%CI) | Case | Control | *P*_replication_^a^ | OR (95%CI) | *P*_combined_^b^ | OR (95%CI) |
| 1 | rs4394137 | 5 | *LOC100128783* | C/T | 0.271 | 0.231 | 1.2×10^-6^ | 1.24 (1.14-1.35) | 0.254 | 0.252 | 0.76 | 1.01 (0.94-1.09) | 1.0×10^-3^ | 1.10 (1.04-1.16) |
| 2 | rs2233434 | 6 | *NFKBIE* | G/A | 0.254 | 0.216 | 2.2×10^-6^ | 1.24 (1.13-1.35) | 0.245 | 0.215 | 4.2×10^-6^ | 1.19 (1.10-1.27) | 4.1×10^-11^ | 1.21 (1.14-1.28) |
| 3 | rs723184 | 22 | *MN1* | T/C | 0.775 | 0.737 | 3.3×10^-6^ | 1.23 (1.13-1.34) | 0.761 | 0.753 | 0.25 | 1.04 (0.97-1.12) | 1.2×10^-4^ | 1.12 (1.06-1.18) |
| 4 | rs10168266 | 2 | *STAT4* | A/G | 0.306 | 0.266 | 5.2×10^-6^ | 1.22 (1.12-1.32) | 0.299 | 0.275 | 8.0×10^-4^ | 1.12 (1.05-1.20) | 3.2×10^-8^ | 1.16 (1.10-1.22) |
| 5 | rs1750734 | 10 | *CUGBP2* | G/A | 0.890 | 0.862 | 8.6×10^-6^ | 1.30 (1.16-1.46) | 0.868 | 0.866 | 0.65 | 1.02 (0.93-1.12) | 1.7×10^-3^ | 1.12 (1.04-1.21) |
| 6 | rs4520564 | 11 | *TRPC6* | T/C | 0.344 | 0.305 | 1.0×10^-5^ | 1.20 (1.10-1.30) | 0.316 | 0.324 | 0.30 | 0.97 (0.90-1.03) | 0.050 | 1.05 (1.00-1.11) |
| 7 | rs6500380 | 16 | *LONP2* | C/T | 0.136 | 0.109 | 1.3×10^-5^ | 1.29 (1.15-1.44) | 0.121 | 0.124 | 0.53 | 0.97 (0.88-1.07) | 0.027 | 1.09 (1.01-1.17) |
| 8 | rs10165970 | 2 | *NPAS2* | A/G | 0.164 | 0.135 | 1.5×10^-5^ | 1.26 (1.13-1.40) | 0.154 | 0.151 | 0.70 | 1.02 (0.93-1.11) | 2.6×10^-3^ | 1.11 (1.04-1.18) |
| 9 | rs7598681 | 2 | *LOC647077* | G/A | 0.196 | 0.164 | 1.6×10^-5^ | 1.24 (1.12-1.36) | 0.178 | 0.180 | 0.71 | 0.98 (0.91-1.07) | 0.016 | 1.08 (1.01-1.15) |
| 10 | rs6571713 | 14 | *LOC122589* | T/C | 0.932 | 0.909 | 1.6×10^-5^ | 1.36 (1.18-1.57) | 0.921 | 0.920 | 0.78 | 1.02 (0.91-1.14) | 3.1×10^-3^ | 1.14 (1.05-1.25) |
| 11 | rs1117769 | 7 | *FOXK1* | A/G | 0.115 | 0.0900 | 1.8×10^-5^ | 1.31 (1.16-1.49) | 0.109 | 0.102 | 0.17 | 1.07 (0.97-1.18) | 1.5×10^-4^ | 1.16 (1.08-1.26) |
| 12 | rs13128082 | 4 | *LOC100131639* | A/G | 0.936 | 0.914 | 1.9×10^-5^ | 1.38 (1.19-1.59) | 0.925 | 0.927 | 0.57 | 0.97 (0.86-1.09) | 0.021 | 1.11 (1.02-1.22) |
| 13 | rs3783762 | 14 | *PRKCH* | T/C | 0.856 | 0.826 | 2.1×10^-5^ | 1.25 (1.13-1.39) | 0.840 | 0.835 | 0.36 | 1.04 (0.96-1.13) | 7.2×10^-4^ | 1.12 (1.05-1.20) |
| 14 | rs17293874 | 4 | *FSTL5* | G/A | 0.215 | 0.182 | 2.2×10^-5^ | 1.22 (1.11-1.34) | 0.207 | 0.201 | 0.36 | 1.04 (0.96-1.12) | 7.6×10^-4^ | 1.11 (1.04-1.17) |
| 15 | rs10503139 | 18 | *CCDC102B* | A/C | 0.847 | 0.817 | 2.4×10^-5^ | 1.24 (1.12-1.38) | 0.827 | 0.823 | 0.52 | 1.03 (0.95-1.11) | 1.6×10^-3^ | 1.11 (1.04-1.18) |
| 16 | rs707097 | 2 | *GALNT13* | T/C | 0.791 | 0.757 | 2.4×10^-5^ | 1.21 (1.11-1.33) | 0.770 | 0.770 | 0.99 | 1.00 (0.93-1.08) | 7.6×10^-3^ | 1.08 (1.02-1.14) |
| 17 | rs9533469 | 13 | *ENOX1* | T/C | 0.115 | 0.0907 | 2.4×10^-5^ | 1.30 (1.15-1.47) | 0.108 | 0.0978 | 0.037 | 1.11 (1.01-1.23) | 1.7×10^-5^ | 1.19 (1.10-1.28) |
| 18 | rs7526484 | 1 | *WNT4* | A/G | 0.170 | 0.141 | 2.6×10^-5^ | 1.25 (1.13-1.38) | 0.157 | 0.151 | 0.34 | 1.04 (0.96-1.13) | 6.8×10^-4^ | 1.12 (1.05-1.20) |
| 19 | rs679670 | 6 | *TNFAIP3* | G/A | 0.180 | 0.151 | 2.8×10^-5^ | 1.24 (1.12-1.37) | 0.168 | 0.154 | 0.015 | 1.11 (1.02-1.20) | 6.1×10^-6^ | 1.16 (1.09-1.24) |
| 20 | rs6877546 | 5 | *PHF15* | C/T | 0.592 | 0.552 | 2.9×10^-5^ | 1.18 (1.09-1.27) | 0.570 | 0.567 | 0.66 | 1.01 (0.95-1.08) | 2.9×10^-3^ | 1.08 (1.03-1.13) |
| 21 | rs4648356 | 1 | *LOC390988* | C/A | 0.524 | 0.484 | 3.3×10^-5^ | 1.17 (1.09-1.27) | 0.508 | 0.494 | 0.071 | 1.06 (1.00-1.13) | 5.1×10^-5^ | 1.10 (1.05-1.16) |
| 22 | rs8060376 | 16 | *LOC390735* | T/C | 0.750 | 0.714 | 3.8×10^-5^ | 1.20 (1.10-1.30) | 0.726 | 0.724 | 0.71 | 1.01 (0.95-1.09) | 3.8×10^-3^ | 1.08 (1.03-1.14) |
| 23 | rs9878356 | 3 | *NLGN1* | C/T | 0.0914 | 0.0700 | 3.9×10^-5^ | 1.34 (1.17-1.53) | 0.0826 | 0.0767 | 0.15 | 1.09 (0.97-1.21) | 1.9×10^-4^ | 1.18 (1.08-1.29) |
| 24 | rs7965910 | 12 | *SCYL2* | C/A | 0.970 | 0.954 | 4.2×10^-5^ | 1.53 (1.25-1.88) | 0.951 | 0.957 | 0.084 | 0.88 (0.76-1.02) | 0.25 | 1.07 (0.95-1.21) |
| 25 | rs8024138 | 15 | *VPS18* | T/C | 0.115 | 0.0913 | 4.3×10^-5^ | 1.29 (1.14-1.46) | 0.112 | 0.101 | 0.021 | 1.12 (1.02-1.24) | 1.3×10^-5^ | 1.19 (1.10-1.28) |
| 26 | rs4350841 | 21 | *PFKL* | C/T | 0.416 | 0.378 | 4.4×10^-5^ | 1.17 (1.09-1.27) | 0.411 | 0.389 | 3.6×10^-3^ | 1.10 (1.03-1.17) | 1.4×10^-6^ | 1.13 (1.07-1.18) |
| 27 | rs6549565 | 3 | *KBTBD8* | A/G | 0.122 | 0.0978 | 4.4×10^-5^ | 1.28 (1.14-1.45) | 0.105 | 0.111 | 0.19 | 0.93 (0.85-1.03) | 0.12 | 1.06 (0.98-1.15) |
| 28 | rs12459507 | 19 | *DOT1L* | T/G | 0.750 | 0.715 | 4.5×10^-5^ | 1.20 (1.10-1.30) | 0.732 | 0.729 | 0.67 | 1.02 (0.95-1.09) | 3.5×10^-3^ | 1.08 (1.03-1.14) |
| 29 | rs174306 | 22 | *CECR2* | G/A | 0.437 | 0.398 | 4.7×10^-5^ | 1.17 (1.09-1.26) | 0.412 | 0.410 | 0.83 | 1.01 (0.95-1.07) | 5.7×10^-3^ | 1.07 (1.02-1.12) |
| 30 | rs3125734 | 10 | *RTKN2* | T/C | 0.125 | 0.101 | 4.8×10^-5^ | 1.27 (1.13-1.43) | 0.129 | 0.110 | 1.4×10^-4^ | 1.20 (1.09-1.31) | 3.7×10^-8^ | 1.23 (1.14-1.32) |
| 31 | rs706622 | 2 | *LOC100130702* | G/T | 0.202 | 0.172 | 4.9×10^-5^ | 1.22 (1.11-1.34) | 0.189 | 0.185 | 0.51 | 1.03 (0.95-1.11) | 2.1×10^-3^ | 1.10 (1.04-1.17) |

a:Cochran-Armitage trend test.

b: Meta-analysis of the GWAS and the replication study-1 (Mantel-Haenszel method).

GWAS, genome-wide association study; OR, odds ratio.
