# Supplementary material for: Functional Variants in NFKBIE and RTKN2 Involved in Activation of the NF-κB Pathway Are Associated with Rheumatoid Arthritis in Japanese
Source: PLoS Genet. 2012 Sep 13;8(9):e1002949. doi: 10.1371/journal.pgen.1002949 (PMC3441678; doi:10.1371/journal.pgen.1002949)
Supplement: Table S3 — Association analysis of NFKBIE and RTKN2 with autoimmune diseases. (DOC) [file pgen.1002949.s011.doc]

**Table S3. Association analysis of *NFKBIE* and *RTKN2* with autoimmune diseases.**

|  |  |  | Allele | Number of subjects | | Frequency of allele 1 | |  |  |
| --- | --- | --- | --- | --- | --- | --- | --- | --- | --- |
| Diseases | Gene | dbSNP ID | (1/2) | Case | Control | Case | Control | Odds ratio (95% CI) | *P*-value^a^ |
| SLE | *NFKBIE* | rs2233434 | G/A | 656 | 35,340 | 0.222 | 0.215 | 1.04 (0.91-1.19) | 0.54 |
|  | *RTKN2* | rs3125734 | T/C | 655 | 35,349 | 0.124 | 0.108 | 1.16 (0.99-1.37) | 0.073 |
|  |  |  |  |  |  |  |  |  |  |
| Graves’ disease | *NFKBIE* | rs2233434 | G/A | 1,782 | 35,340 | 0.214 | 0.215 | 1.00 (0.92-1.08) | 0.93 |
|  | *RTKN2* | rs3125734 | T/C | 1,780 | 35,349 | 0.130 | 0.108 | 1.24 (1.12-1.37) | 3.4×10^-5^ |

a: Cochran-Armitage trend test.

SLE, Systemic lupus erythematosus.
