# Supplementary material for: Functional Variants in NFKBIE and RTKN2 Involved in Activation of the NF-κB Pathway Are Associated with Rheumatoid Arthritis in Japanese
Source: PLoS Genet. 2012 Sep 13;8(9):e1002949. doi: 10.1371/journal.pgen.1002949 (PMC3441678; doi:10.1371/journal.pgen.1002949)
Supplement: Table S4 — Association analysis of nsSNPs with RA. (DOC) [file pgen.1002949.s012.doc]

**Table S4. Association analysis of nsSNPs with RA.**

|  |  | Allele | Number of subjects | | Frequency of allele 1 | |  |  |
| --- | --- | --- | --- | --- | --- | --- | --- | --- |
| Gene | dbSNP ID | (1/2) | Case | Control^b^ | Case | Control | Odds ratio (95% CI) | *P*-value^a^ |
| *NFKBIE* | rs2233433 | T/C | 2,297 | 3,247 | 0.254 | 0.215 | 1.25 (1.14-1.36) | 1.3×10^-6^ |
|  | rs2233434 | G/A | 2,299 | 3,270 | 0.255 | 0.217 | 1.24 (1.13-1.35) | 4.0×10^-6^ |
|  |  |  |  |  |  |  |  |  |
| *RTKN2* | rs61850830 | T/C | 2,292 | 3,283 | 0.123 | 0.103 | 1.21 (1.08-1.37) | 1.4×10^-3^ |
|  | rs3125734 | T/C | 2,296 | 3,281 | 0.125 | 0.100 | 1.29 (1.15-1.45) | 2.3×10^-5^ |

a: Cochran-Armitage trend test.

b: A part of control samples used in the GWAS were genotyped (*n* = 3,290)
