# Supplementary material for: Functional Variants in NFKBIE and RTKN2 Involved in Activation of the NF-κB Pathway Are Associated with Rheumatoid Arthritis in Japanese
Source: PLoS Genet. 2012 Sep 13;8(9):e1002949. doi: 10.1371/journal.pgen.1002949 (PMC3441678; doi:10.1371/journal.pgen.1002949)
Supplement: Table S5 — Haplotype association study of nsSNPs in NFKBIE. (DOC) [file pgen.1002949.s013.doc]

**Table S5. Haplotype association study of nsSNPs in *NFKBIE*.**

|  | nsSNP | | Frequency | |  |  |
| --- | --- | --- | --- | --- | --- | --- |
| haplotype | rs2233434 | rs2233433 | Case | Control | Odds ratio (95% CI) | *P*-value |
| haplotype-1 | A | C | 0.745 | 0.779 | 0.83 (0.76-0.91) | 2.6×10^-5^ |
| haplotype-2 | G | T | 0.254 | 0.210 | 1.28 (1.17-1.40) | 5.3×10^-8^ |
