# Supplementary material for: Functional Variants in NFKBIE and RTKN2 Involved in Activation of the NF-κB Pathway Are Associated with Rheumatoid Arthritis in Japanese
Source: PLoS Genet. 2012 Sep 13;8(9):e1002949. doi: 10.1371/journal.pgen.1002949 (PMC3441678; doi:10.1371/journal.pgen.1002949)
Supplement: Table S6 — Haplotype association study of nsSNPs in RTKN2. (DOC) [file pgen.1002949.s014.doc]

**Table S6. Haplotype association study of nsSNPs in *RTKN2*.**

|  | nsSNP | | Frequency | |  |  |
| --- | --- | --- | --- | --- | --- | --- |
| haplotype | rs3125734 | rs61850830 | Case | Control | Odds ratio (95% CI) | *P*-value |
| haplotype-1 | C | C | 0.860 | 0.882 | 0.82 (0.76-0.92) | 7.0×10^-4^ |
| haplotype-2 | T | T | 0.108 | 0.0885 | 1.29 (1.13-1.46) | 5.7×10^-5^ |
| haplotype-3 | C | T | 0.0145 | 0.0179 | 0.83 (0.61-1.12) | 0.16 |
| haplotype-4 | T | C | 0.0169 | 0.0145 | 1.22 (0.90-1.65) | 0.30 |
