# Supplementary material for: Functional Variants in NFKBIE and RTKN2 Involved in Activation of the NF-κB Pathway Are Associated with Rheumatoid Arthritis in Japanese
Source: PLoS Genet. 2012 Sep 13;8(9):e1002949. doi: 10.1371/journal.pgen.1002949 (PMC3441678; doi:10.1371/journal.pgen.1002949)
Supplement: Table S7 — Predicting the effects of nsSNPs on protein function. (DOC) [file pgen.1002949.s015.doc]

**Table S7. Predicting the effects of nsSNPs on protein function.**

| Gene | dbSNP ID | Allele change | Residue change | Position | Polarity | Acidic/Basic | Isoelectric point | SIFT^a^ | PolyPhen^b^ |
| --- | --- | --- | --- | --- | --- | --- | --- | --- | --- |
| *NFKBIE* | rs2233433 | C→T | P [Pro]→L [Leu] | 175 | non-polar→non-polar | neutral→neutral | 6.3→6.0 | tolerated | benign |
|  | rs2233434 | A→G | V [Val]→A [Ala] | 194 | non-polar→non-polar | neutral→neutral | 6.0→6.0 | tolerated | benign |
|  |  |  |  |  |  |  |  |  |  |
| *RTKN2* | rs61850830 | C→T | A [Ala]→T [Thr] | 288 | non-polar→polar | neutral→neutral | 6.0→6.2 | tolerated | benign |
|  | rs3125734 | C→T | R[Arg]→H [His] | 462 | polar→polar | basic→basic | 10→7.6 | tolerated | benign |

a: SIFT: Sort Intolerant from Tolerant (http://sift.jcvi.org/).

b: Polyphen: Polymorphism Phenotype (http://genetics.bwh.harvard.edu/pph/).
