# Supplementary material for: Functional Variants in NFKBIE and RTKN2 Involved in Activation of the NF-κB Pathway Are Associated with Rheumatoid Arthritis in Japanese
Source: PLoS Genet. 2012 Sep 13;8(9):e1002949. doi: 10.1371/journal.pgen.1002949 (PMC3441678; doi:10.1371/journal.pgen.1002949)
Supplement: Table S8 — Association analysis of candidate rSNPs with RA. (DOC) [file pgen.1002949.s016.doc]

**Table S8. Association analysis of candidate rSNPs with RA.**

a: Cochran-Armitage trend test.

b: A part of control samples used in the GWAS were genotyped (*n* = 3,290)

|  |  | Allele | Number of subject | | Frequency of allele 1 | |  |  |
| --- | --- | --- | --- | --- | --- | --- | --- | --- |
| Gene | dbSNP ID | (1/2) | Case | Control^b^ | Case | Control | Odds ratio (95% CI) | *P*-value^a^ |
| *NFKBIE* | rs2233424 | T/C | 2,298 | 3,264 | 0.255 | 0.216 | 1.24 (1.14-1.36) | 2.3×10^-6^ |
|  |  |  |  |  |  |  |  |  |
| *RTKN2* | rs61852964 | T/G | 2,297 | 3,262 | 0.121 | 0.0995 | 1.25 (1.11-1.41) | 2.8×10^-3^ |
|  | rs12248974 | G/A | 2,300 | 3,286 | 0.123 | 0.100 | 1.27 (1.12-1.43) | 9.4×10^-5^ |
