# Supplementary material for: Functional Variants in NFKBIE and RTKN2 Involved in Activation of the NF-κB Pathway Are Associated with Rheumatoid Arthritis in Japanese
Source: PLoS Genet. 2012 Sep 13;8(9):e1002949. doi: 10.1371/journal.pgen.1002949 (PMC3441678; doi:10.1371/journal.pgen.1002949)
Supplement: Table S9 — Haplotype association study of candidate causal SNPs in NFKBIE. (DOC) [file pgen.1002949.s017.doc]

**Table S9. Haplotype association study of candidate causal SNPs in *NFKBIE*.**

|  | rs2233434 | rs2233433 | rs2233424 | Frequency | |  |  |
| --- | --- | --- | --- | --- | --- | --- | --- |
| haplotype | The landmark SNP nsSNP | nsSNP | rSNP | Case | Control | Odds ratio (95% CI) | *P*-value |
| haplotype-1 | A | C | C | 0.745 | 0.775 | 0.83 (0.78-0.93) | 2.0×10^-4^ |
| haplotype-2 | G | T | T | 0.253 | 0.207 | 1.30 (1.19-1.42) | 7.8×10^-9^ |
