# Supplementary material for: Functional Variants in NFKBIE and RTKN2 Involved in Activation of the NF-κB Pathway Are Associated with Rheumatoid Arthritis in Japanese
Source: PLoS Genet. 2012 Sep 13;8(9):e1002949. doi: 10.1371/journal.pgen.1002949 (PMC3441678; doi:10.1371/journal.pgen.1002949)
Supplement: Table S10 — Haplotype association study of candidate causal SNPs in RTKN2. (DOC) [file pgen.1002949.s018.doc]

**Table S10. Haplotype association study of candidate causal SNPs in *RTKN2*.**

|  | rs12248974 | rs3125734 | rs61852964 | Frequency | |  |  |
| --- | --- | --- | --- | --- | --- | --- | --- |
| haplotype | rSNP | The landmark SNP  nsSNP | rSNP | Case | Control | Odds ratio (95% CI) | *P*-value |
| haplotype-1 | A | C | G | 0.859 | 0.882 | 0.82 (0.73-0.91) | 4.0×10^-4^ |
| haplotype-2 | G | T | T | 0.105 | 0.0827 | 1.30 (1.14-1.47) | 6.0×10^-5^ |
| haplotype-3 | G | T | G | 0.0168 | 0.0135 | 1.22 (0.90-1.66) | 0.16 |
| haplotype-4 | A | C | T | 0.0140 | 0.0146 | 0.93 (0.68-1.28) | 0.79 |
