# Supplementary material for: Functional Variants in NFKBIE and RTKN2 Involved in Activation of the NF-κB Pathway Are Associated with Rheumatoid Arthritis in Japanese
Source: PLoS Genet. 2012 Sep 13;8(9):e1002949. doi: 10.1371/journal.pgen.1002949 (PMC3441678; doi:10.1371/journal.pgen.1002949)
Supplement: Table S11 — The conditional haplotype-based association analysis of candidate causal SNPs in RTKN2. (DOC) [file pgen.1002949.s019.doc]

**Table S11. The conditional haplotype-based association analysis of candidate causal SNPs in *RTKN2*.**

|  |  |  | *P*-values conditioned on | | |
| --- | --- | --- | --- | --- | --- |
| dbSNP ID |  | Original *P*-value | rs12248974 | rs3125734 | rs61852964 |
| rs61852964 | rSNP | 2.8×10^-4^ | 0.67 | 0.79 | - |
| rs3125734 | the landmark SNP, nsSNP | 2.3×10^-5^ | n/a | - | 0.055 |
| rs12248974 | rSNP | 9.4×10^-5^ | - | n/a | 0.28 |

n/a: not assessed. Conditional analysis was not performed due to the high LD between the SNPs.
