# Supplementary material for: Functional Variants in NFKBIE and RTKN2 Involved in Activation of the NF-κB Pathway Are Associated with Rheumatoid Arthritis in Japanese
Source: PLoS Genet. 2012 Sep 13;8(9):e1002949. doi: 10.1371/journal.pgen.1002949 (PMC3441678; doi:10.1371/journal.pgen.1002949)
Supplement: Table S12 — Probes and Primers used for TaqMan assays. (DOC) [file pgen.1002949.s020.doc]

**Table S12. Probes and Primers used for TaqMan assays.**

| Assays | Gene | SNP | Probe sets (5’-3’) | | Primer sets (5’-3’) | |
| --- | --- | --- | --- | --- | --- | --- |
| Genotyping assays | *NFKBIE* | rs2233434 | VIC | VIC- CTGTGGTTCCTTGACG -MGB | Forward | CCATCCGCGTCTTCCTTCT |
|  |  |  | FAM | FAM- TGTGGTTCCTTGGCGG -MGB | Reverse | GCCCTGCACCCATCCTC |
|  |  | rs2233433 | VIC | VIC- CTCGGCTCCAGCCT -MGB | Forward | CGGAGGAGAGCCAGTACGACT |
|  |  |  | FAM | FAM- TCGGCTCTAGCCTC -MGB | Reverse | TTCCTTCTCCTGTGGTTCCTTG |
|  |  | rs2233424 | VIC | VIC- CAGCTTGTACAGACAATTA -MGB | Forward | CCCATCCCATCGCCTACAT |
|  |  |  | FAM | FAM- AGCTTGTACGGACAATTA -MGB | Reverse | GTCCCGGATCATTCATTTGTTT |
|  | *RTKN2* | rs3125734 | VIC | VIC- TTCTTCACGCTGACCAA -MGB | Forward | GGCCCAAGGAGGTGGTAAG |
|  |  |  | FAM | FAM- ATTCTTCATGCTGACCAA -MGB | Reverse | GCATTGATTCACCTATGAAACTTGA |
|  |  | rs61850830 | VIC | VIC- CTGCAAATGTATCCTC -MGB | Forward | CAGAAGGTGAAACAACTGCCTGTATA |
|  |  |  | FAM | FAM- CTGCAAATGCATCCT -MGB | Reverse | CATACAAGCTGGCTGGGCA |
|  |  | rs12248974 | VIC | VIC- AAACACCACGTGGTCC -MGB | Forward | TCAGAAATCACCACTAAAGAACTTACTCA |
|  |  |  | FAM | FAM- CAAACACCATGTGGTCC -MGB | Reverse | TCATTGCGTGTATGTATACATGTACACA |
|  |  | rs61852964 | VIC | VIC- AGCTTTCCCCGCGGC -MGB | Forward | GTCCCGGACAGGGATGTG |
|  |  |  | FAM | FAM- AAGCTTTCCCCGAGGC -MGB | Reverse | TCCAGCTACGATCACTTTGGAA |
