# Supplementary material for: Functional Variants in NFKBIE and RTKN2 Involved in Activation of the NF-κB Pathway Are Associated with Rheumatoid Arthritis in Japanese
Source: PLoS Genet. 2012 Sep 13;8(9):e1002949. doi: 10.1371/journal.pgen.1002949 (PMC3441678; doi:10.1371/journal.pgen.1002949)
Supplement: Table S13 — Primers used for DNA re-sequencing. (DOC) [file pgen.1002949.s021.doc]

**Table S13. Primers used for DNA re-sequencing.**

| Gene | Exon | Primer sequences (5’-3’) ^a^ | |
| --- | --- | --- | --- |
|  |  | Forward | Reverse |
| *NFKBIE* | 1 | GATAAATGGGCAGGGGATAG | GCAGCGAGGACAAGGTTC |
|  | 1 | GACTCCAAGCCTACGCAGA | AGGAGCCATAGGTGGAATCA |
|  | 1 | CCAGTACGACTCTGGCATTG | AAGATGCTGGCTGGGTTAAG |
|  | 2 | GGACTACTTCGGCAGCTTTC | CTCTTGTCACCAGGCTGTGTA |
|  | 3 | TCACCTTGCTTCCAGAATTG | TCTTCTCAGCAGGCAGTAGG |
|  | 4 | GACCCCTGAAGCATGAAAGT | GCACAGAGTGGATGAGATGC |
|  | 5 | CACTCATGGAATTGCTGCTT | TTATAGGCCTCACCACACCA |
|  | 6 | AACAAACAACTCCAGGTCCA | ACAGGCTTCACCCAGGATAC |
| *RTKN2* | 1 | GCGGGCTTTTCAAATCTTCC | CTGGGTTTCTTGGGAATCG |
|  | 2 | AGGGATGCTATTAAACTTCGT | TCAGCTGCTACTTCAGTGT |
|  | 3 | CGGGAGTGGCTCCAGATATT | TGCACTAGGGAGAAAACTGTAGA |
|  | 4 | ACAGACATTTACTCAGAATGGCA | CCTAGCACTGGTTCCTATCA |
|  | 5 | ACTGTAAGACTTAAGGGTTACAG | GAGAAATGGACTTCTTCTGAACA |
|  | 6 | AGCCATCCTTATGGGTGTGA | GATACCTCAGTTGAGTAAATGTC |
|  | 7 | TGCTGGATCTTGTCCTCTC | CCACTAAGCCTTGCAAGCA |
|  | 8 | TGGCACTGGTATTTGGGTAG | AGGTAGCGCCTTAGCCATCA |
|  | 9 | GGATTTGGGGCAATTAACCT | GCACAGACAAAAACAAACTTCC |
|  | 10 | CTCATGTAATTTAGCATAAGTGC | GAAGCATCAGTGCATTGATG |
|  | 11 | CAGAACTGGACTTTGAAATTATGG | CTATTAAACATGAGTTCCTTAGTC |
|  | 12 | ACAAGTGAACGTTTTCCTTATCA | TCACTTACATTCTGCAAATCAGG |

a:Primers **were designed based** on human sequences obtained from the **NCBI reference sequence** database (*NFKBIE* region, NT_007592.14; *RTKN2* region, NT_008583.16)
