# Supplementary material for: Functional Variants in NFKBIE and RTKN2 Involved in Activation of the NF-κB Pathway Are Associated with Rheumatoid Arthritis in Japanese
Source: PLoS Genet. 2012 Sep 13;8(9):e1002949. doi: 10.1371/journal.pgen.1002949 (PMC3441678; doi:10.1371/journal.pgen.1002949)
Supplement: Table S14 — Primers used for construction of expression vectors. (DOC) [file pgen.1002949.s022.doc]

**Table S14. Primers used for consruction of expression vectors.**

| Gene | Primer sequences (5’-3’)^a^ | |
| --- | --- | --- |
|  | Forward | Reverse |
| *NFKBIE* | CACCATGTCGGAGGCGCGGAAG | GTCGGTACACAGCAGCAGTTTCCCTG |
| *RTKN2* | CACCATGGAGGGGCCGAGCCTG | TACTTGTGCCTGCAGCCATG |

a:Primers **were designed based** on human sequences obtained from the **NCBI reference sequence** database.

(*NFKBIE* region, NM_004556.2; *RTKN2* region, NM_145307.2)
