# Supplementary material for: Functional Variants in NFKBIE and RTKN2 Involved in Activation of the NF-κB Pathway Are Associated with Rheumatoid Arthritis in Japanese
Source: PLoS Genet. 2012 Sep 13;8(9):e1002949. doi: 10.1371/journal.pgen.1002949 (PMC3441678; doi:10.1371/journal.pgen.1002949)
Supplement: Table S15 — Oligonucleotides used for EMSAs and Luciferase assays. (DOC) [file pgen.1002949.s023.doc]

**Table S15. Oligonucleotides used for EMSAs and Luciferase assays.**

| Gene | SNP | Non-risk allele | Sequence (5’-3’)^a^ | Risk allele | Sequence (5’-3’) |
| --- | --- | --- | --- | --- | --- |
| *NFKBIE* | rs2233437 | A | GGCAGGCCCTGGGCCAGGCAGTGCTTTGCTG | G | GGCAGGCCCTGGGCCGGGCAGTGCTTTGCTG |
|  | rs3799962 | G | GTCTAAGGGGAAAGGGACTCAAAGTTTCCAC | A | GTCTAAGGGGAAAGGAACTCAAAGTTTCCAC |
|  | rs3799963 | G | AGGAAAGTGCCCCCTGCTCTCTGGGGACTCG | C | AGGAAAGTGCCCCCTCCTCTCTGGGGACTCG |
|  | rs730775 | G | TTAAGTGAGGGAGTGGGACACGACAGTTAGT | A | TTAAGTGAGGGAGTGAGACACGACAGTTAGT |
|  | rs2233434 | A | TCCTGTGGTTCCTTGACGGGTCCCGGAGGAT | G | TCCTGTGGTTCCTTGGCGGGTCCCGGAGGAT |
|  | rs2233433 | G | GAGGGCCCGGAGGCTGGAGCCGAGGTGGACT | A | GAGGGCCCGGAGGCTAGAGCCGAGGTGGACT |
|  | rs2233424 | C | TATCTATTAATTGTCCGTACAAGCTGGCATC | T | TATCTATTAATTGTCTGTACAAGCTGGCATC |
|  | rs79082484 | T | GCTCTAAGCAATAGGTGGACAGAGAGCCACC | C | GCTCTAAGCAATAGGCGGACAGAGAGCCACC |
|  | rs77986492 | C | CAGGAAGGCACTTGCCCAAGGTCACATAACT | T | CAGGAAGGCACTTGCTCAAGGTCACATAACT |
|  | rs3734709 | G | GTACGCCTGCTCGTCGGCCTTCTGGGCCGCG | A | GTACGCCTGCTCGTCAGCCTTCTGGGCCGCG |
|  | rs74950428 | G | TCCTGAGGGAGGGTAGTGGTCAAGGTGCCTG | C | TCCTGAGGGAGGGTACTGGTCAAGGTGCCTG |
|  | rs9688508 | A | AGGAATGAACATAAGAAGGGGGCTGACTTAC | G | AGGAATGAACATAAGGAGGGGGCTGACTTAC |
|  | rs78740068 | C | TTCCCAGCTCAAGCCCTCCCCAAGCCCTCCC | T | TTCCCAGCTCAAGCCTTCCCCAAGCCCTCCC |
|  | rs167772 | T | TCAGTATGGTTCGGCTCTCACCTGGTTCATG | C | TCAGTATGGTTCGGCCCTCACCTGGTTCATG |
| *RTKN2* | rs12784499 | A | GGACAATGTAGACACACCAATTCACCTGACA | G | GGACAATGTAGACACGCCAATTCACCTGACA |
|  | rs12248974 | A | TTTTTGGGGGACCACATGGTGTTTGGTTACA | G | TTTTTGGGGGACCACGTGGTGTTTGGTTACA |
|  | rs76457651 | C | AGTACCAAGAGCTCTCGGTGCTTAAATACCT | T | AGTACCAAGAGCTCTTGGTGCTTAAATACCT |
|  | rs35576264 | C | GGAGTCTTGACCAGCCTGGCCAACATGGGGA | T | GGAGTCTTGACCAGCTTGGCCAACATGGGGA |
|  | rs7904465 | A | TACCTTTTTTTTTTTAAGACAGCGTCTTGCT | G | TACCTTTTTTTTTTTGAGACAGCGTCTTGCT |
|  | rs3864793 | C | TGAATTTATTTTCAACAGTTTCATCTTTTAT | T | TGAATTTATTTTCAATAGTTTCATCTTTTAT |
|  | rs1864836 | G | CACGCGCAGTGGGCGGGCCTTGCGCTCTGCA | C | CACGCGCAGTGGGCGCGCCTTGCGCTCTGCA |
|  | rs61852964 | G | GGAGTGGGAGGGGCCGCGGGGAAAGCTTCGG | T | GGAGTGGGAGGGGCCTCGGGGAAAGCTTCGG |
|  | rs4979765 | C | CCAATGCACATCTCTCTCTTATGCTACCACT | G | CCAATGCACATCTCTGTCTTATGCTACCACT |
|  | rs4979766 | C | TGTGGTATAATTATCCCCTACTCAGTATGAG | T | TGTGGTATAATTATCTCCTACTCAGTATGAG |

a:Oligonucleotides **were designed based** on human sequences obtained from the **NCBI reference sequence** database (*NFKBIE* region, NT_007592.14; *RTKN2* region, NT_008583.16)
